# Glycosylation of a Fasciclin-Like Arabinogalactan-Protein (SOS5) Mediates Root Growth and Seed Mucilage Adherence via a Cell Wall Receptor-Like Kinase (FEI1/FEI2) Pathway in Arabidopsis

**DOI:** 10.1371/journal.pone.0145092

**Published:** 2016-01-05

**Authors:** Debarati Basu, Lu Tian, Tayler Debrosse, Emily Poirier, Kirk Emch, Hayley Herock, Andrew Travers, Allan M. Showalter

**Affiliations:** Molecular and Cellular Biology Program, Department of Environmental and Plant Biology, Ohio University, Athens, Ohio 45701–2979, United States of America; Institute of Genetics and Developmental Biology, Chinese Academy of Sciences, CHINA

## Abstract

Fundamental processes that underpin plant growth and development depend crucially on the action and assembly of the cell wall, a dynamic structure that changes in response to both developmental and environmental cues. While much is known about cell wall structure and biosynthesis, much less is known about the functions of the individual wall components, particularly with respect to their potential roles in cellular signaling. Loss-of-function mutants of two arabinogalactan-protein (AGP)-specific galactosyltransferases namely, *GALT2* and *GALT5*, confer pleiotropic growth and development phenotypes indicating the important contributions of carbohydrate moieties towards AGP function. Notably, *galt2galt5* double mutants displayed impaired root growth and root tip swelling in response to salt, likely as a result of decreased cellulose synthesis. These mutants phenocopy a salt-overly sensitive mutant called *sos5*, which lacks a fasciclin-like AGP (SOS5/FLA4) as well as a *fei1fei2* double mutant, which lacks two cell wall-associated leucine-rich repeat receptor-like kinases. Additionally, *galt2gal5* as well as *sos5* and *fei2* showed reduced seed mucilage adherence. Quintuple *galt2galt5sos5fei1fei2* mutants were produced and provided evidence that these genes act in a single, linear genetic pathway. Further genetic and biochemical analysis of the quintuple mutant demonstrated involvement of these genes with the interplay between cellulose biosynthesis and two plant growth regulators, ethylene and ABA, in modulating root cell wall integrity.

## Introduction

Growing plant cells control the biogenesis, deposition, and remodeling of the cell wall. The plant cell wall is a dynamic and complex structure composed of carbohydrates and proteins that play crucial roles in all aspects of plant life. To avoid loss of integrity, which may lead to growth cessation, continuous cellular surveillance is required to sense cell wall perturbations and coordinate cell wall performance with the internal growth machinery [1]. Mounting evidence indicates that a dedicated plant cell wall integrity (CWI) maintenance mechanism exists in plants [2]. This mechanism monitors and maintains the functional integrity of the wall during different biological processes, including exposure to abiotic and biotic stress [3], [4]. While our understanding of the mechanisms involved in biosynthesis of cell wall polymers have increased significantly, current understanding of the components and mechanisms involved in the perception and regulation of processes maintaining CWI is limited.

Plant cell walls are composite structures composed of cellulose and matrix polysaccharides such as hemicelluloses and pectins [5]. Besides the polysaccharides, cell wall proteins represent another important component of plant cell walls and are involved in wall structure, support, signaling, and interactions with other wall components and with the plasma membrane [6]. Arabinogalactan-proteins (AGPs) are one such cell wall glycoprotein family that is implicated to function in various aspects of plant growth and development, including root growth and development, somatic embryogenesis, hormone responses, xylem differentiation, pollen tube growth and guidance, programmed cell death, cell expansion, salt tolerance, and cellular signaling [7–11]. Evidence for these functions is circumstantial, indirect, or based on genetic mutant analysis. While these functional implications speak to the importance of AGPs in plants, there are virtually no studies that delve into the biochemical mechanism of action for these proposed AGP functions.

Fasciclin-like arabinogalactan-proteins (FLAs) are a subfamily of AGPs characterized by the presence of one or two fasciclin (Fas 1) domains, which are cell adhesion domains [12]. FLAs are frequently predicted to have a glycosylphosphatidylinositol (GPI) anchor, which would allow for their localization to the plasma membrane, making them ideal candidates for signal perception and transduction. Indeed, FLAs are implicated in cell wall biosynthesis, cell wall remodeling, and signaling [9]. For example, the *FLA11* and *FLA12* genes are preferentially expressed in secondary cell wall forming cells [13], [14], similar to their orthologues from other plant species [15]. Moreover, *fla11fla12* double mutants display a substantial reduction in cellulose content accompanied by reduced tensile strength and tensile modulus of elasticity [15]. Another fasciclin-like AGP, namely *FLA3*, is specifically expressed in pollen grains and tubes and involved in microspore development, possibly by participating in cellulose deposition [16]. *SOS5* (salt overly sensitive 5), also known as *FLA4*, represents a FLA implicated in wall remodeling and signaling and is involved in root growth under elevated salt or sucrose containing media [17]. Xu et al. [18] demonstrated that SOS5 acts upstream of cell wall deposition in a non-additive genetic pathway by interacting with FEI1/FEI2, two cell wall leucine-rich repeat receptor-like kinases (RLKs), based on the identical phenotypes displayed by single, double and triple mutants, namely, *sos5*, *fei1fei2*, and *sos5fei1fei2* loss-of-function mutants. These mutants demonstrated reduced cell elongation accompanied by impaired anisotropic growth (i.e., root tip swelling) when grown under the restrictive conditions of elevated salt or sucrose. Furthermore, the swollen root tip phenotype was reversed in all these mutants by blocking ethylene biosynthesis, but not ethylene perception [18]. The recent characterization of two genetic mutants namely, *galt2* and *galt5*, which encode two galactosyltransferase (GALT) enzymes responsible for adding the first galactose onto AGP protein backbones displayed identical phenotypes as observed in *sos5*, *fei1fei2*, and *sos5fei1fei2* mutants [19]. Since these *galt* mutants have reduced glycosylation of AGPs [19], it is hypothesized that glycosylation of SOS5 is required for its function in the SOS5/FEI1-FEI2 pathway to signal normal root growth under non-permissive conditions.

Harpaz-Saad et al. [20] also demonstrated that SOS5 and FEI2 play a novel and non-conditional role in formation of transverse cellulosic rays deposited across the inner layer of seed coat mucilage. Malformation of these rays in these mutants coincides with an increase in the solubility of the pectinaceous component of seed coat mucilage. Disruption of *GALT2* and *GALT5* in *galt2galt5* double mutants resulted in an identical seed coat mucilage phenotype [19]. Based on the above information, it is hypothesized that glycosylation of SOS5 catalyzed by GALT2 and GALT5 is essential for cellular signaling of normal root growth, cellulosic ray formation, and extrusion of seed coat mucilage in Arabidopsis. This paper provides support for this hypothesis by generating and functionally characterizing quintuple mutant plants (*galt2galt5sos5fei1fei2*) along with other related genetic mutants, such as *galt2galt5* double mutants and *sos5fei1fei2* triple mutants.

## Materials and Methods

### Plant material

The Columbia (Col-0) ecotype of Arabidopsis thaliana was used in this study. The *galt2-1* and *galt5-1* mutant lines used in this study were previously characterized by Basu et al. [19], [21]. The *sos5fei1fei2* triple mutant was the kind gift of Dr. Joseph Keiber, University of North Carolina Chapel Hill, North Carolina, USA. The quintuple mutant was generated by crossing a *galt2galt5* (*galt2-1* and *galt5-1*) homozygous double mutant with a *sos5fei1fei2* homozygous triple mutant. Heterozygous quintuple mutants were obtained in the F1 generation and selfed to produce an F2 population, which was selfed to obtain homozygous quintuple mutants. Mutant progeny, including quintuple mutants, were identified by PCR genotyping using gene-specific and T-DNA primers listed in S1 Table. All the insertion sites for all the mutants were confirmed by DNA sequencing of PCR-amplified products. The *cesa5* mutant and *cesa5sos5* double mutant were the kind gifts of Dr. George Haughn, University of British Columbia, Vancouver, Canada. The *cesa2cesa5cesa9* triple mutant was the kind gift of Dr. Seth DeBolt, University of Kentucky, Lexington, Kentucky, USA.

### Seed sterilization

Wild type (WT) and mutant seeds were surface-sterilized by washing in a 95% ethanol solution for 5 min followed by a 5 min wash in a 30% bleach with 0.1% Tween 20 solution, and then rinsed seven times with sterile water. Seeds were sown on 1X MS nutrient medium containing 1% sucrose and 0.6% agar. For stratification treatment, seeds were stratified at 4°C in the dark for 3 d and were subsequently transferred to a 16-h-light/8-h-dark cycle at 22°C at 110 μmol m^−2^ s^−1^ irradiance.

### RT and qRT-PCR analysis

For seed expression, Arabidopsis siliques were staged by marking flowers following anthesis and sampled at 4, 7, 10, and 14 DPA. For differential expression analysis in tissues/organs, 30 d old plants were used. Roots were obtained from 14 d old seedlings grown in liquid culture [22]. Total RNA was isolated using the RNeasy Plant Mini Kit (Qiagen) and the RNase-Free DNase Set (Qiagen). First-strand cDNA synthesis was performed from 2 μg of total RNA using oligo-dT (IDT) and Go Script reverse transcriptase (Promega, Madison, WI, USA). RT-PCR was performed using OneTaq DNA polymerase (New England Biolabs) and gene-specific primers (S1 Table). The number of amplification cycles was kept at 26 to ensure accurate evaluation and quantification of transcript levels before the reaction has reached saturation. For qRT-PCR, the cDNAs were amplified using Brilliant II SYBR Green qRT-PCR Master Mix with ROX (Agilent Technologies, La Jolla, CA, USA) in an MX3000P real-time PCR instrument (Agilent Technologies). The qRT-PCR conditions were optimized, and reactions were performed in triplicate. The gene-specific primers used are listed in S1 Table. Expression levels were calculated using the comparative CT method, which involves normalizing against the geometric mean of two housekeeping genes, Ubiquitin 10 (*UBQ10*, *At4g05320*) and Protein phosphatase 2 (*PP2A*, *At1g13320*), for each tissue type and glyceraldehyde-3-phosphate dehydrogenase (GAPC, *At3g04120*) for seed [23], [24].

### Cellulose synthesis assays

Cellulose synthesis was determined by [^14^C]Glc labeling as described by Fagard et al. [25] and Xu et al. [18] with the following modifications. WT and mutant seedlings were grown on MS plates with 0% sucrose for 4 d and then transferred to MS medium containing either 4.5% sucrose or 100 mM NaCl for 5 d. Root tips (~1.5 cm) were cut and washed three times with 3 mL of glucose-free MS medium. Forty root tips were incubated in 1 mL of MS medium containing 0.1 μCi/mL [^14^C]Glc for 1 h in the dark at 22°C in glass tubes. Subsequently, these roots were washed three times with 1 mL of glucose-free MS medium. Next, the roots were extracted three times with 1 mL of boiling absolute ethanol for 20 min, and aliquots were collected (ethanol-soluble fraction). Roots were then resuspended in 1 mL of chloroform:methanol (1:1, v/v) for 20 min at 45°C, and finally resuspended in 1 mL of acetone for 15 min at room temperature with gentle shaking. The remaining material was resuspended in 500 μL of an acetic acid: nitric acid: water solution (8:1:2, v/v/v) for 1 h in a boiling-water bath. Acid-soluble material and acid-insoluble material were separated by glass microfiber filters (GF/A; 2.5 cm diameter; Whatman), after which the filters were washed with 3 mL of water. The acid wash and water wash constitute the acid-soluble fraction. The filters yield the acid-insoluble fraction. The amount of label in each fraction was determined with a Model LS6500 multipurpose scintillation counter (Beckman) using liquid scintillation fluid (Scintiverse BD cocktail; SX 18–4; Fisher). The percentage of label incorporation was expressed as 100X the ratio of the amount of label in each fraction to the total amount of label (ethanol plus acid-soluble plus acid-insoluble fractions).

### Germination assay

Mature seeds were placed on filter paper in petri dishes and moistened with an aqueous solution of 12–21% (w/v) PEG 8000. The seeds were cold treated at 4°C for 4 days in the dark and then grown at 22°C in 110 μE constant light for another 5 days. Germination was scored by radical protrusion.

### Crystalline cellulose determination

Crystalline cellulose levels were determined based on the Updegraff method [26]. Five milligrams of seeds were frozen in liquid nitrogen, ground using a mortal and pestle, and dried at 50°C overnight. Ground seeds were treated with 1 mL of the acetic-nitric acid reagent (stock solution, 150 mL of 80% [v/v] glacial acetic acid diluted with water and 15 mL of [70%] concentrated nitric acid) and vortexed. Samples were heated at 100°C for 1 h, centrifuged for 5 min at 3,000 rpm, and washed. Samples were then treated with 1 mL 72% (w/v) H_2_SO_4_, vortexed, incubated at room temperature for 1 h, centrifuged for 5 min at 10,000 rpm, and diluted 10 times in distilled water. One hundred microliters of diluted sample were treated with 200 μL of cold, freshly prepared anthrone reagent (0.2% [w/v] anthrone [Sigma-Aldrich] in concentrated H_2_SO_4_) and vortexed. Anthrone mixtures were incubated for 15 min at 100°C, and duplicate samples were measured two times at A_620_ in a GENESYS^™^ 10S UV-Vis Spectrophotometer. A standard curve was prepared by dilution of a dextrose stock solution. Total amounts of cellulose were calculated per weight of dry seed mass.

### Cell wall preparation

One-hundred milligrams of WT and mutant seeds were extracted sequentially with 0.2% ammonium oxalate, 0.2N sodium hydroxide, and 2N sodium hydroxide for 1 h each with vigorous shaking at 37°C. Both sodium hydroxide extractions were neutralized with acetic acid. Total sugar (nmol/mg seed) was determined with a phenol-sulfuric assay based on Dubois et al. [27]. Briefly, 200 μL of resuspended extract was incubated with 100 μl freshly made 5% (v/v) aqueous phenol and 1 mL concentrated sulfuric acid for 2 h at 30°C. Absorbance was detected at 500 nm against glucose standards of 0, 10, 50, 100, 150, 200, 250, and 300 μM final concentrations for which a linear response curve was obtained.

### Seed staining and visualization

WT and mutant seeds were prehydrated in water and stained with 0.01% ruthenium red. Pontamine Fast Scarlet S4B and calcofluor staining were performed as described by Willats et al. [28] and Harpaz-Saad et al. [20]. Imaging was done using a Zeiss LSM 510 confocal microscope. For calcofluor staining, excitation was at 405 nm and emission detection was at 421–558 nm. For pontamine Fast Scarlet S4B staining, a 561 nm diode laser was used for excitation followed by emission detection at 605–640 nm. Cellulosic rays were measured from the base of the ray to the tip as described by Ben-Tov et al. [29].

### Phenotypic analysis

For seedling growth in salt, 7 d old WT and mutants seedlings were transferred to MS medium containing 1% agar and 100 mM or 150 mM NaCl. Root length was determined on low-magnification (×10) digital images captured using a CCD camera and image analysis freeware (ImageJ; http://rsb.info.nih.gov/ij/). For analysis of salt hypersensitivity of the mutant plants, root growth was also monitored using a root bending assay [30], and images were taken with a Nikon SMZ1500 stereomicroscope coupled with a CCD Infinity 2 camera and analysis software.

### Phloroglucinol staining

Phloroglucinol staining for lignin was performed as previously described by Cano-Delgado et al. [31]. Seedlings were fixed in a solution of three parts ethanol to one part acetic acid for 15 min and then stained in a 2% phloroglucinol-HCl solution for 5 min.

### Cell number and length measurements

For determination of root meristematic cell number and root cell length, five day old WT and mutant seedlings were transferred from MS plates to MS plates supplemented with 100 mM NaCl or 4.5% sucrose for another five days. Seedlings were then stained with propidium iodide 1μg/ml for 10 min and washed three times with 1X PBS. Confocal stacks of roots were obtained with a Zeiss LSM 510 confocal microscope. Roots were excited at 488 nm, and the signal was detected with a band-pass 625–655 nm filter. Epidermal cell length was averaged from at least 10 cells per root at a distance of one cm from the root tips. At least five roots were examined for each treatment, and cell lengths were measured using ImageJ software.

### Statistical analysis

Statistical analyses were performed using SPSS software version 18 (IBM). One-way ANOVA was used to analyze phenotypic differences, treating genotype and condition together as a single factor. Tukey’s post hoc tests were used to deduce statistically significant means (P < 0.05), as indicated by different letters in the relevant figures and tables.

## Results

### Expression patterns of *GALT2*, *GALT5*, *SOS5*, *FEI1*, and *FEI2*

**Fig 1:**
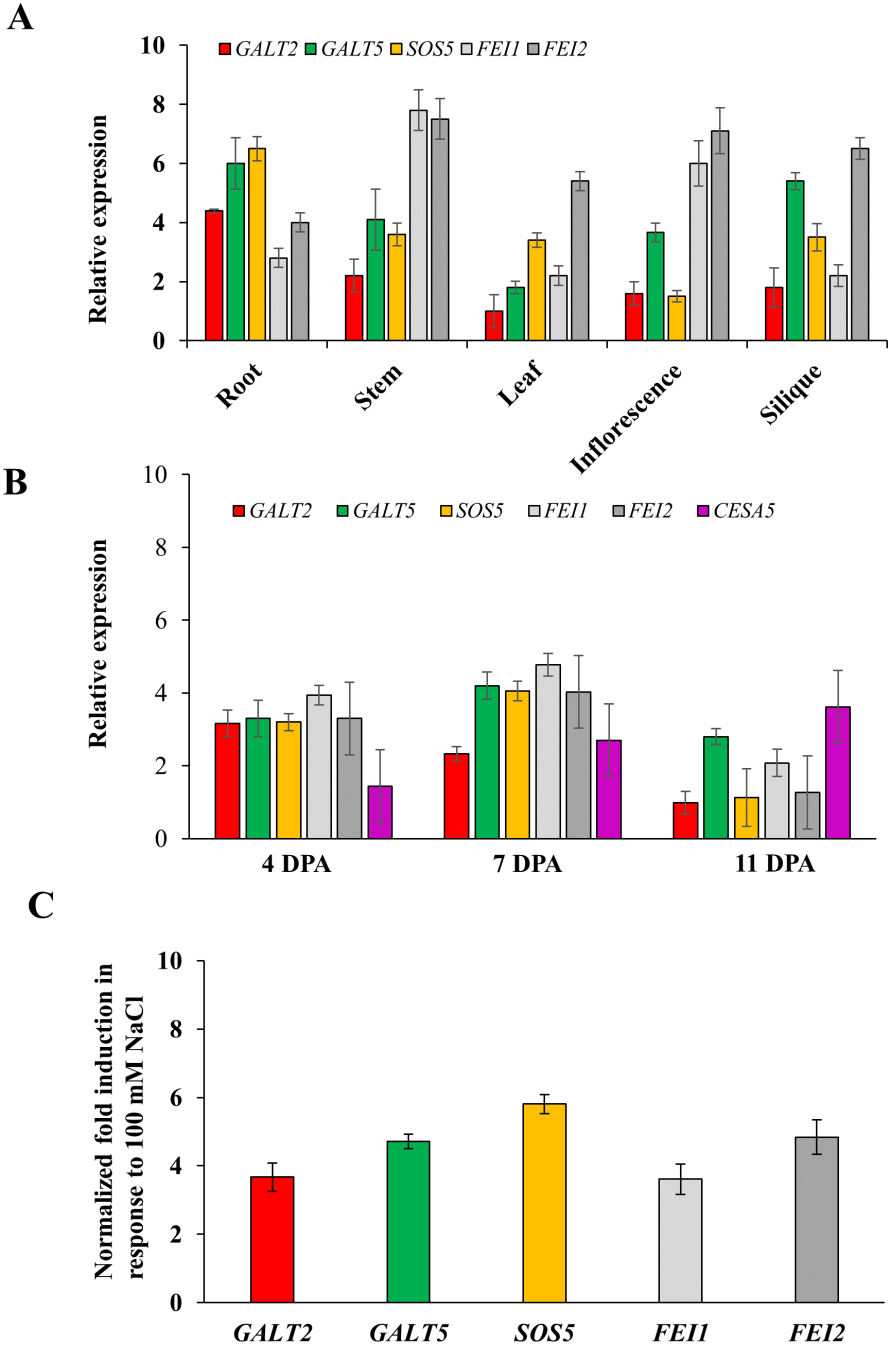
Transcript profiling of *GALT2*, *GALT5*, *SOS5*, *FEI1*, and *FEI2* genes in different organs/tissues and in response to salt treatment. (**A**) qRT-PCR analysis of the five genes in different plant organs/tissues. Expression levels are the mean ± SE of three technical replicates relative to the *Ubiquitin 10* (*UBQ10*) reference gene. The expression value of *GALT2* in leaf was considered as 1. **(B)** Expression analysis of the five genes during the course of seed development. Total RNA was isolated from seeds 4, 7, and 11 DPA. Relative expression was normalized using *GAPC* as a reference gene. The expression value of *GALT2* at 11 DPA was considered as 1. **C.** Induction of transcript abundance of *GALT2*, *GALT5*, *SOS5*, *FEI1*, and *FEI2* in response to salt treatment. Total RNA was extracted from root tips of 250 WT and mutant seedlings grown in MS media and in MS media supplemented with 100 mM NaCl for 7d. Experiments in (**B**) and (**C**) are the mean ± SE of three technical replicates.

RNA transcript abundance profiles of *GALT2*, *GALT5*, *FEI1*, and *FEI2* were examined in various vegetative and floral tissues using Arabidopsis PlaNet, a publicly available dataset (S1 Fig). SOS5 was not included in this analysis due to its absence from the ATH1 Affymetrix chip. Analysis revealed ubiquitous but distinct expression patterns for the four genes throughout plant development [32]. *In silico* analysis revealed generally higher transcript abundance of *GALT2*, *GALT5*, *FEI1*, and *FEI2* in primary roots, early stages of floral development, and in siliques during early stages of seed development. Since *SOS5* was not present on the Affymetrix ATH1 array, the relative expression levels of the five genes were further assessed using quantitative RT-PCR (qRT-PCR) and showed expression patterns consistent with the microarray analysis (Fig 1A). Additionally, transcriptome analyses using RNA extracted from laser-capture dissected seed coat tissue indicated that the transcript levels of *GALT2*, *GALT5*, *FEI1*, *FEI2*, and *SOS5* are high in the seed coat during early embryogenesis (S2 Fig; http://seedgenenetwork.net/arabidopsis; http://bar.utoronto.ca/efp_seedcoat/cgi-bin/efpWeb.cgi) [33], [34]. Further qRT-PCR experiments were performed to assess the expression pattern of the *GALT2*, *GALT5*, *SOS5*, *FEI1*, and *FEI2* genes during the course of seed development (Fig 1B). The expression of *GALT2* and *GALT5*, like *SOS5*, *FEI1*, and *FEI2* was highest at 4–7 DPA, which precedes and coincides with peak production of seed coat mucilage at 7 DPA [35–39]. In contrast, CESA5 was most highly expressed at 11 DPA. Although *FEI1* was expressed more than *FEI2*, *fei1* mutants did not exhibit the aberrant seed coat mucilage phenotype displayed by *fei2* mutants [18]. In addition, the observed expression patterns were similar to that observed for *SOS5*, *CESA5*, *FEI1*, *FEI2*, *GALT2* and *GALT5* using microarray analysis of seed coat development [34]. Furthermore, co-expression analysis using the GeneCAT tool (http://genecat.mpg.de) indicated expression of *FEI1* and *FEI2* is tightly correlated with the expression of AGP-specific glycosyltransferases as well as numerous candidate AGPs (S2 Table) [40]. Collectively, these data indicate the concerted involvement of these five genes in root tip development and seed coat mucilage formation. To assess their roles in salt stress, the transcript abundance of these five genes in root tips were determined by growing wild type (WT) plants in MS supplemented with 100 mM NaCl. All five genes were induced at least three-fold in response to this salt treatment (Fig 1C). It should, however, be noted that the *SOS5* expression data presented here differed somewhat from that previously reported by Shi et al. [17] in terms of organ-specific and salt-induced expression. Such discrepancies may be attributed to their use of northern blotting with a non-specific fasciclin-based hybridization probe, analysis of different portions of the plant organs, and different salt-induction conditions.

### Quintuple mutants display cell expansion defects

**Fig 2:**
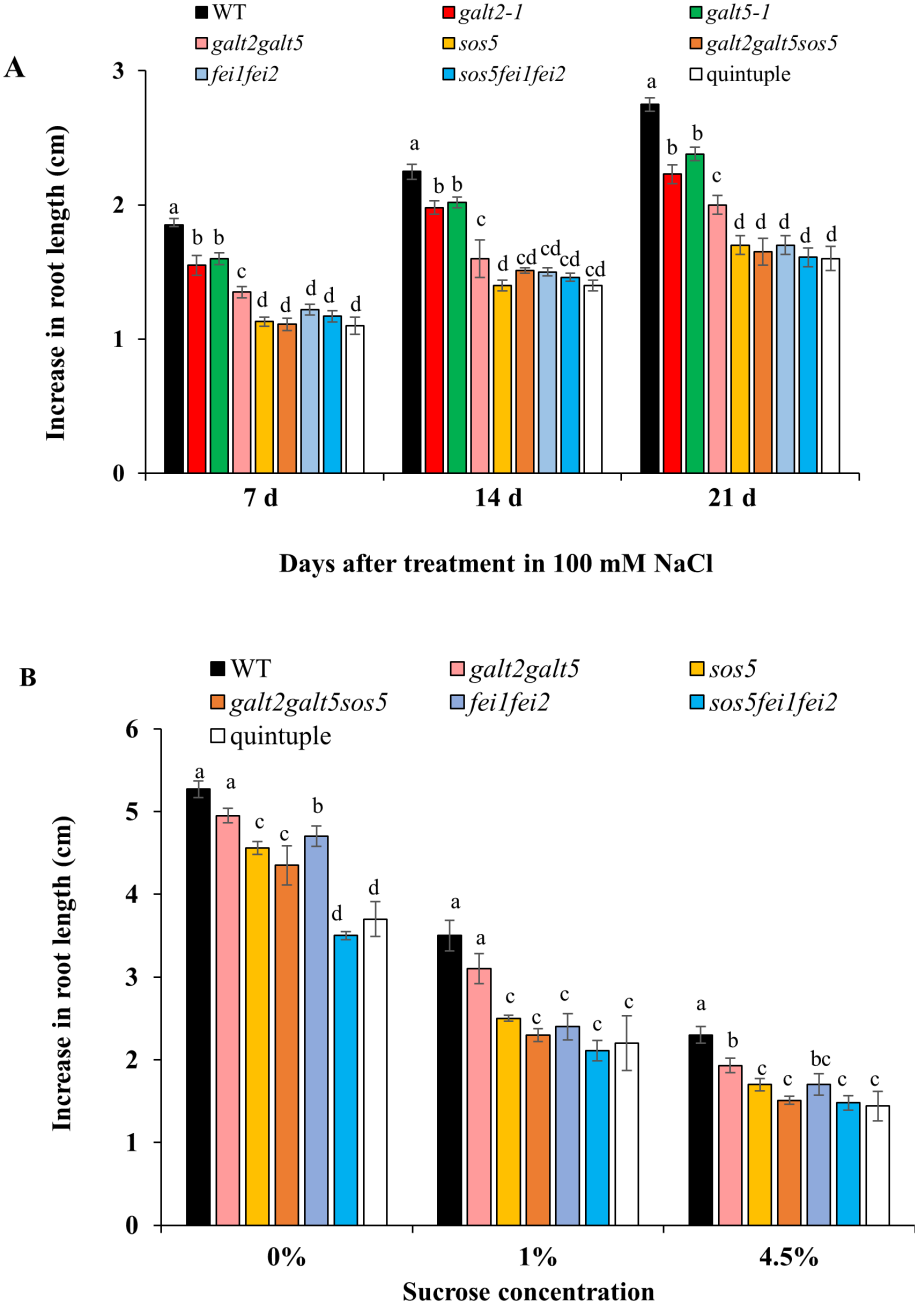
Quintuple mutants exhibit reduced root elongation in response to NaCl and sucrose. **(A)** WT and mutant seedlings were grown on MS medium containing 1% sucrose for 5 d and then transferred to MS medium containing 100 mM NaCl. **(B)** WT and mutant seedlings were grown on MS medium containing 0% sucrose for 5 d and then transferred to MS medium containing 4.5% sucrose. Quantification of root elongation after transfer to non-permissive conditions was recorded after 7, 14, and 21 d. Data are means ± SE; n ≥ 25. Different letters indicate statistically significant differences (P < 0.05) between means.

**Fig 3:**
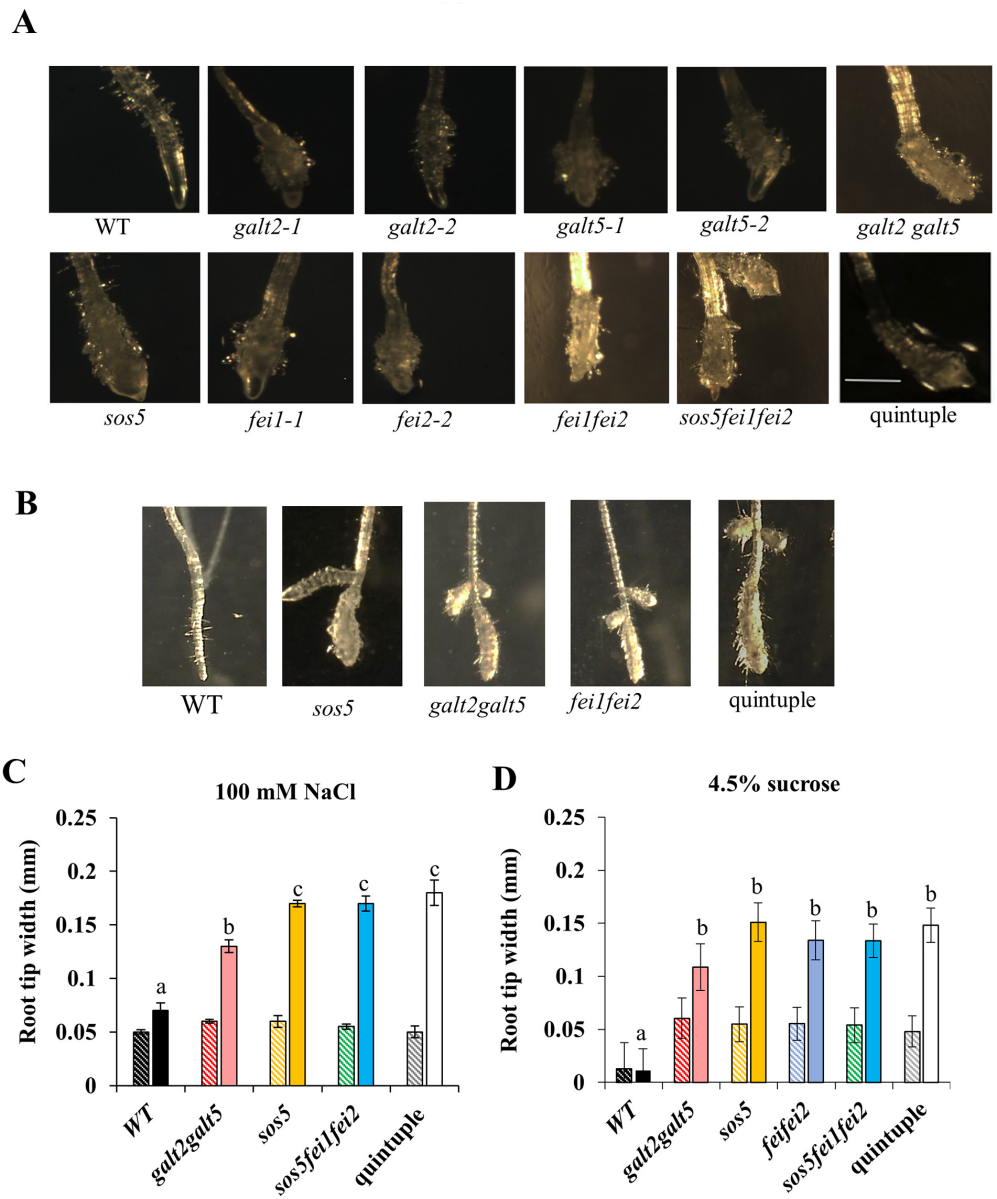
Quintuple mutants display a conditional root anisotropic growth defect (i.e., root swelling). (**A**) Root tips of WT and mutant seedlings four days after transfer from MS medium containing 1% sucrose to MS medium containing 100 mM NaCl as imaged by dark field microscopy. (**B**) Root tips of WT and mutant seedlings four days after transfer from MS medium containing 0% sucrose to MS medium containing 4.5% sucrose as imaged by dark field microscopy. (**C**) Graphical representation of root tip width in response to 100 mM NaCl. (**D**) Graphical representation of root tip width in response to 4.5% sucrose. The stripped bars indicate seedlings grown on control unsupplemented MS plates. Quantitation of root tip width was measured at the level of the youngest root hair using ImageJ software. Values are the means (n>15) ± SE. Different letters indicate statistically significant differences (P < 0.05) between means.

Roots of *sos5* and *fei1fei2* behaved similarly to *galt2galt5* in response to 100 mM NaCl [19]. Thus, a quintuple mutant was generated by crossing *sos5fei1fei2* triple mutants with *galt2galt5* double mutants to test for genetic interaction among *GALT2*, *GALT5*, *SOS5*, *FEI1*, and *FEI2*. RT-PCR analysis confirmed the quintuple mutant was a functional knock-out of all five genes (S3 Fig). The quintuple mutant displayed decreased root elongation and root tip swelling compared to WT plants, and phenocopied both parental lines, *galt2galt5* and *sos5fei1fei2*, in response to elevated salt and sucrose, however, *galt2galt5* showed less severe root growth defects which can be attributed to GALT gene redundancy (Figs 2 and 3). The increased diameter and reduced elongation observed in quintuple mutant roots provided quantitative evidence that anisotropic expansion is defective in the quintuple mutant root cells similar to *sos5fei1fei2* and *galt2galt5*, although *galt2galt5* displayed less intense root swelling defects.

Like roots, hypocotyls of etiolated seedlings are composed of cells that primarily undergo longitudinal expansion. Hypocotyls of etiolated quintuple mutant seedlings exhibited significantly wider hypocotyls (S4 Fig).

The number of meristematic cells in roots of *galt2galt5*, *sos5*, *fei1fei2*, and the quintuple mutants showed no significant difference in response to sucrose or salt treatment compared to WT (S5 Fig). In response to 4.5% sucrose, *galt2galt5*, *sos5fei1fei2*, and quintuple mutants displayed a 17%-26% reduction in root epidermal cell length, whereas in response to 100 mM NaCl, they exhibited a 13%-23% reduction in root epidermal cell length. Importantly, quintuple mutants did not exacerbate the anisotropic growth defect of the double or triple mutant hypocotyls or roots, indicating that the same pathway regulates both root and hypocotyl anisotropic growth. Moreover, quintuple mutants, like their parents, did not exhibit root swelling in the presence of elevated concentrations of mannitol, indicating that the effects of sucrose and NaCl were not the result of a response to elevated osmolarity (S6 Fig).

As an additional test of salt sensitivity of the mutants, the root bending assay was used [41–43]. This assay is based on the ability of roots to sense and reorient themselves relative to gravity by means of a differential growth response achieved by cell expansion occurring in the elongation zone [44]. This assay also revealed that the quintuple mutants, like their parental lines, were salt hypersensitive as indicated by the increased root curvature displayed by the mutants due to their delayed reorientation of root growth (S7 Fig).

### The quintuple mutant is defective in cellulose biosynthesis

**Fig 4:**
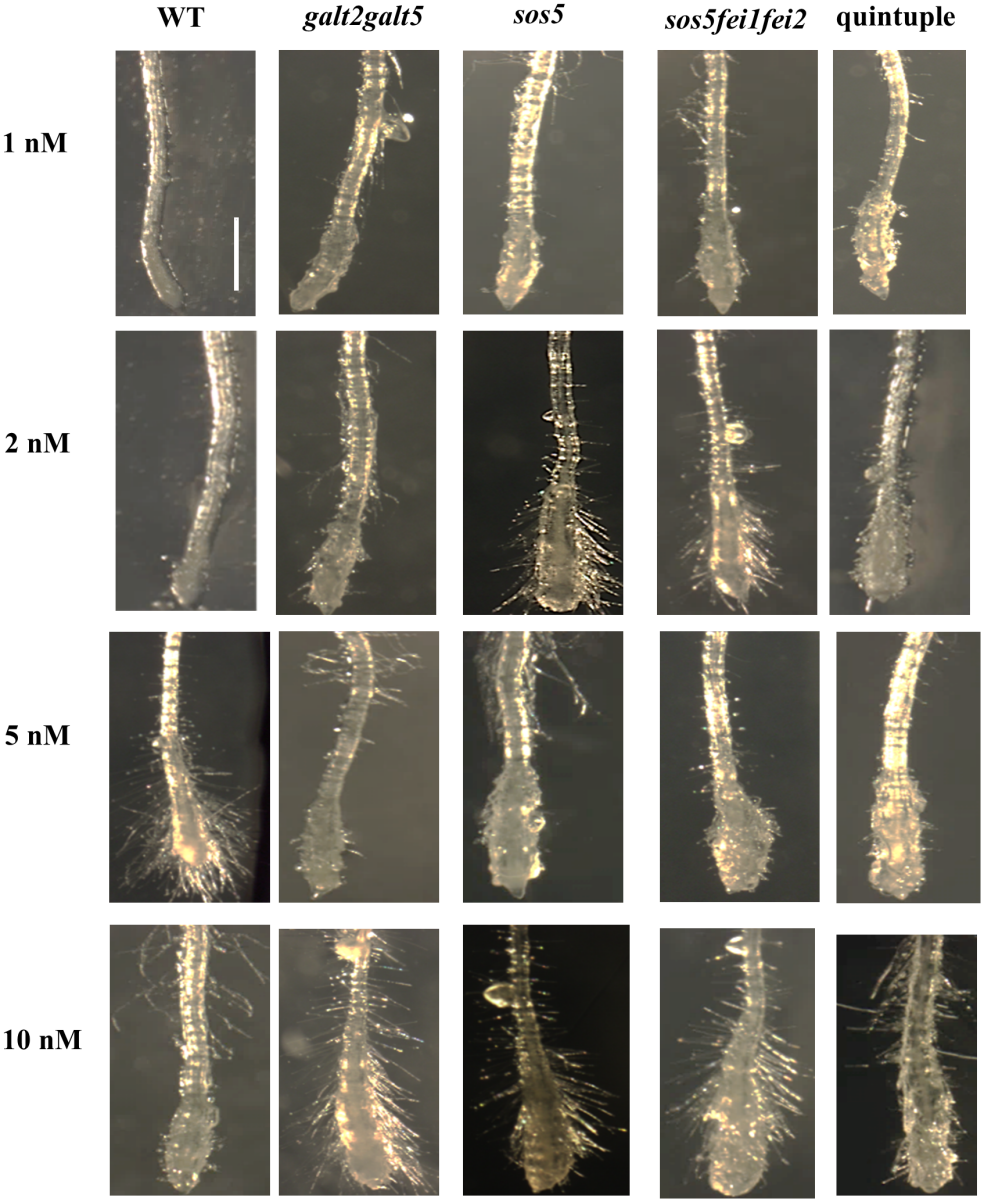
Hypersensitivity of the mutants towards isoxaben. Root tips from WT and mutant seedlings were germinated and grown for five days on MS medium with 1% sucrose and transferred for 72 hours to MS medium supplemented with isoxaben at various concentrations. Root tips were imaged using dark field microscopy. Scale bar = 1 mm.

**Fig 5:**
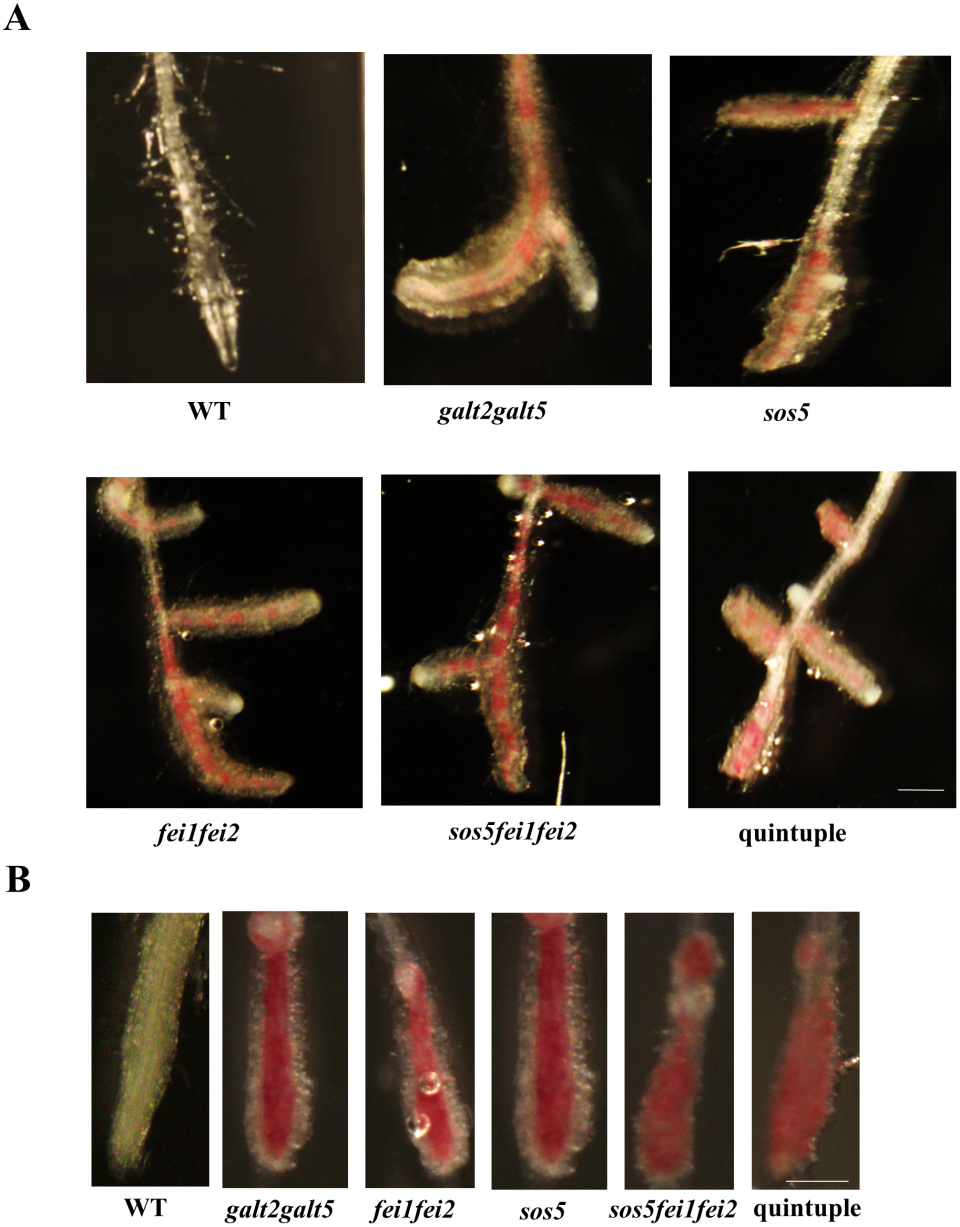
Ectopic lignin deposition in the mutants. WT and mutant seedlings were grown on MS medium containing 0% sucrose for 5 d and then transferred to **(A)** MS medium containing 100 mM NaCl for 7 d or to **(B)** MS medium containing 4.5% sucrose for 7 d. Scale bar = 1 mm.

The altered pattern of root cell expansion in the quintuple mutant likely arises from a cell wall defect. To assess the cell wall properties, the effect of isoxaben, an inhibitor of cellulose synthase, on *fei1fei2*, *galt2galt5*, *sos5*, and the quintuple mutant was examined. Quintuple mutants displayed increased root swelling sensitivity to isoxaben compared to WT seedlings, similar to *fei1fei2*, *sos5*, and *galt2galt5* seedlings (Fig 4). In addition, roots of *fei1fei2*, *sos5fei1fei2*, *galt2galt5*, and quintuple mutant seedlings grown in non-permissive conditions produced ectopic lignin under both elevated salt and sucrose (Fig 5A and 5B). Such phenotypes were previously reported for *fei1fei2* mutants in response to high sucrose [18].

**Fig 6:**
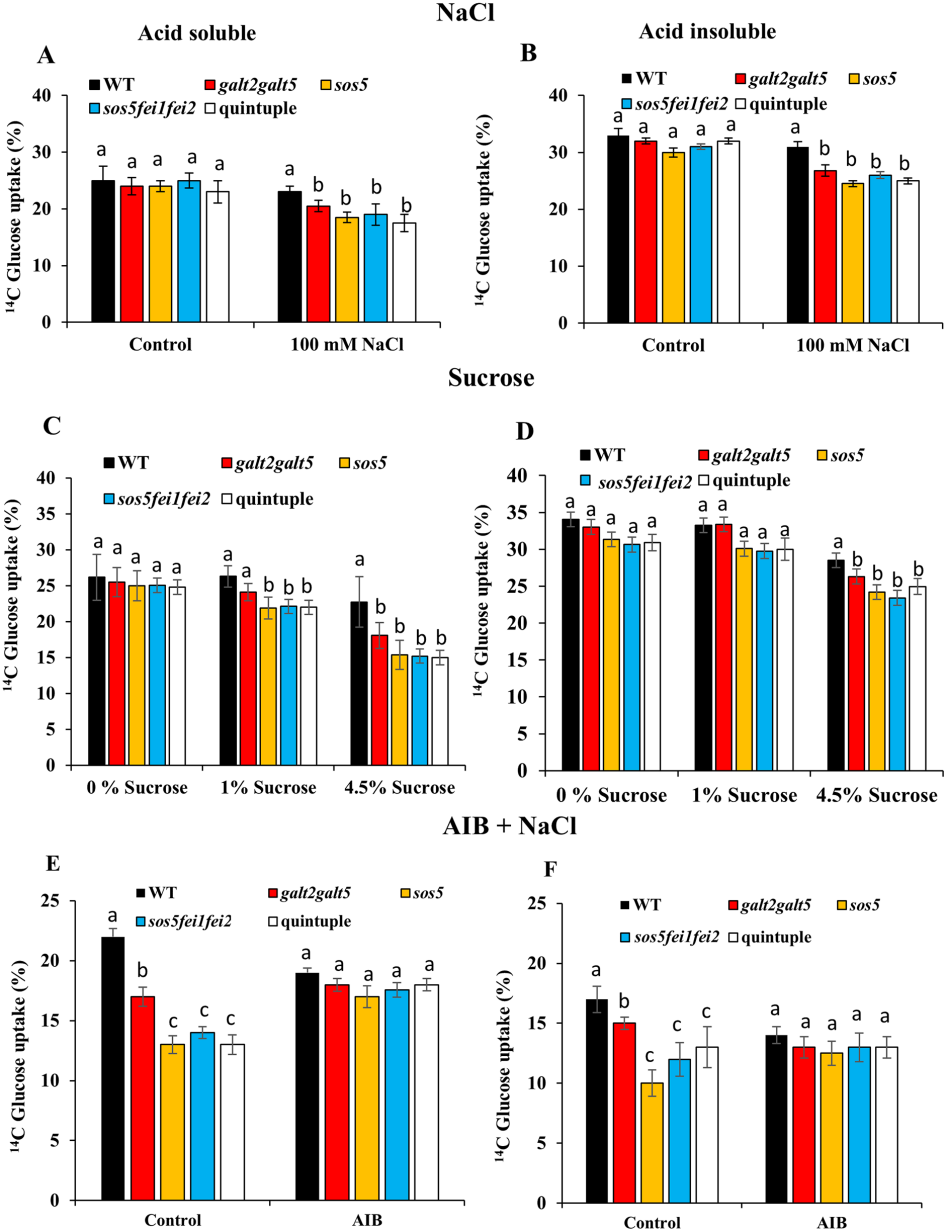
Quintuple mutants display a cellulose deficiency in response to salt. **(A)** and **(B)** Incorporation of [^14^C]Glc into acid-soluble and acid-insoluble fractions from excised root tips from WT and mutant seedlings in response to 100 mM NaCl, (**C**) and **(D)** in response to 4.5% sucrose and **(E)** and **(F)** in response to AIB. Seedlings were grown on 0% sucrose for 5 d and then transferred to the three different treatment conditions for 7d. (**A), (C),** and **(E)** indicate incorporation of [^14^C]Glc into the acid soluble fraction, and (**B), (D),** and **(F)** indicate incorporation of [^14^C]Glc into the acid insoluble fraction. Values are means ± SD from two biological replicates, and the experiments were repeated at least two times with similar results. Different letters indicate statistically significant differences (P < 0.05) between means.

Cellulose synthesis was subsequently analyzed by measuring incorporation of [^14^C] glucose into crystalline and non-crystalline cellulosic cell wall fractions from excised root tips of WT and quintuple mutant seedlings grown under non-permissive conditions. The quintuple mutant, along with *fei1fei2*, *sos5*, *sos5fei1fei2*, and *galt2galt5* displayed a striking reduction in cellulose biosynthesis in response to 100 mM NaCl (i.e., 15–27%) and sucrose treatment (i.e., 11–23%), as measured by the incorporation of radiolabeled [^14^C] UDP glucose into acid-insoluble material (crystalline cellulose) (Fig 6) [45], [46]. It should be noted that the *galt2galt5* mutants were less sensitive to such defects. The acid-soluble material (non-crystalline cellulose and other wall polymers) also exhibited a reduction in response to 100 mM NaCl (i.e., 18–27%) and sucrose treatment (i.e., 13–28%) (Fig 6) [47]. Similar results were previously reported for *fei1fei2* roots [18]. These findings are consistent with impaired cellulose biosynthesis as the presence of ectopic lignin is generally correlated with a decreased level of crystalline cellulose.

### Role of phytohormones in SOS5-FEI1/FEI2 mediated cell expansion

**Fig 7:**
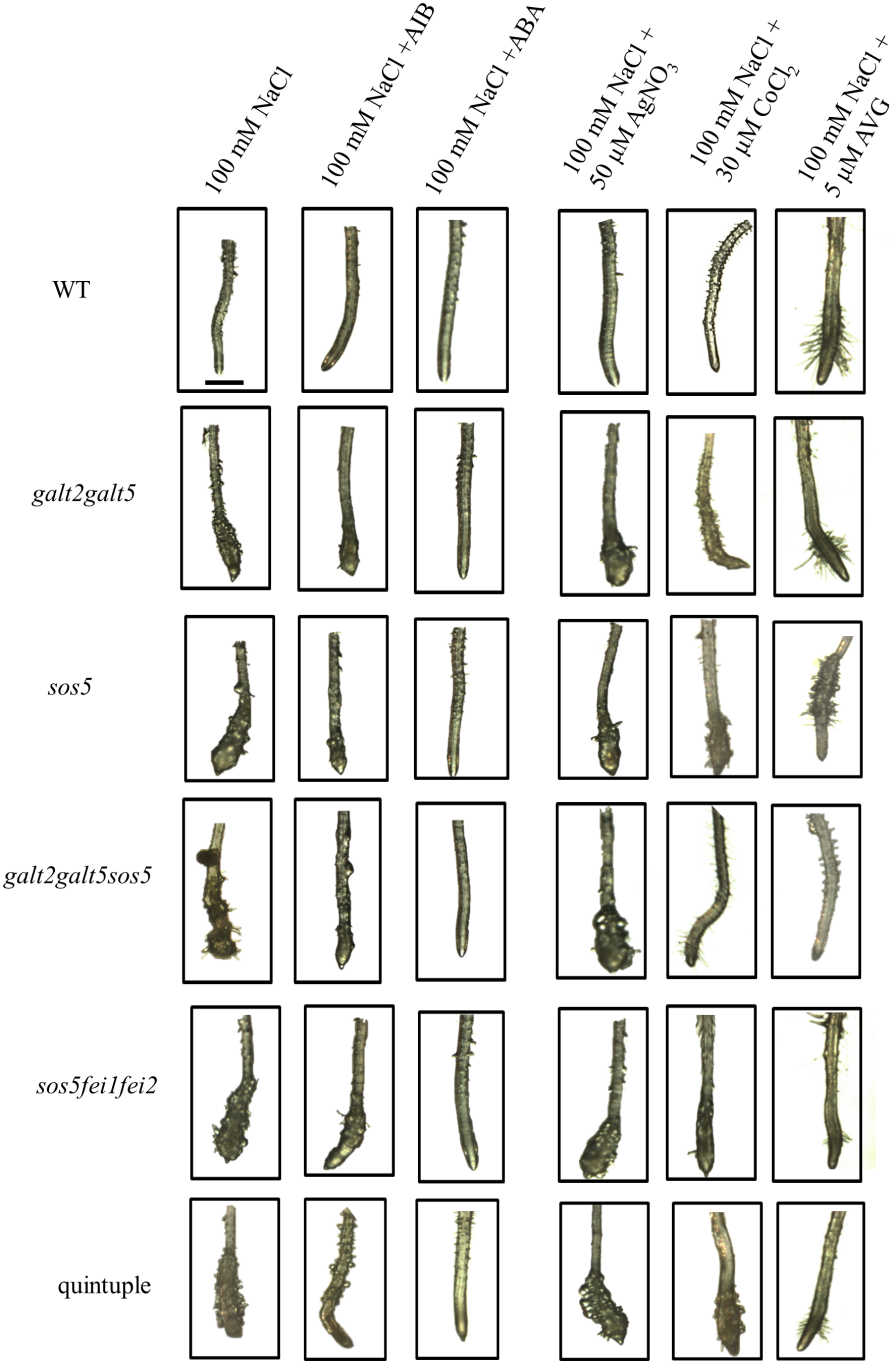
Role of ethylene and ABA on the conditional root swelling phenotype. Images of WT and mutant seedlings grown on MS medium containing 0% sucrose for 4 d and transferred to MS medium containing 100 mM NaCl supplemented with AIB, ABA, AgNO_3_, CoCl_2_, and AVG for 7 d as indicated. Scale bar = 1 mm.

Previous reports demonstrated that inhibition of ethylene biosynthesis or perception can partially revert the swollen phenotypes of certain root morphology mutants, such as *sabre* and *cev1*, thereby indicating ethylene’s crucial role in cell expansion [48], [49]. This information provided the impetus to determine the effect of blocking ethylene signaling or biosynthesis on the swollen root tip phenotype observed in this study. Thus, three ethylene biosynthesis inhibitors, cobalt chloride (CoCl_2_), amino isobutyric (AIB), and aminoethoxyvinyl glycine (AVG), were used. Both CoCl_2_ and AIB block ACC oxidase, but they employ different mechanisms. Co^2+^ ions inhibit ACC oxidase, which is required for the conversion of ACC to ethylene, thus blocking ethylene synthesis, whereas AIB is a structural analog of ACC that blocks ACC oxidase activity by acting as a competitive inhibitor. In contrast, AVG is an inhibitor of pyridoxal phosphate required for ACC synthase (ACS) activity [50]. As AVG, AIB, and CoCl_2_ block ethylene biosynthesis by different mechanisms, it is unlikely that this phenotypic reversion of *fei1fei2*, *sos5*, *galt2galt5* and quintuple mutants is due to off-target effects (Fig 7). Notably, AIB treatment in the presence of salt resulted not only in the reversion of the root swelling phenotype, but also led to reversion to WT levels of cellulose synthesis (Fig 6E and 6F). Silver ions (silver nitrate), which block ethylene perception, had no appreciable effect on the root phenotype of quintuple mutants (Fig 7). Taken together, these results demonstrated that a signaling pathway involving ethylene participates in the reduction of root cell elongation when mutants were subjected to elevated levels of salt and sucrose. In addition, these phenotypes in the quintuple mutant were virtually identical to that of *sos5fei1fei2*, *sos5*, and similar to *galt2galt5* mutants indicating that these genes are likely in the same genetic pathway.

Seifert et al. [51] reported that SOS5 acts synergistically with abscisic acid (ABA) signaling to control root growth. Consequently, the role of ABA was assessed here, and revealed that 5 μM ABA suppresses the swollen root tip phenotype in all mutants (Fig 7). Furthermore, it was observed that similar to the phenotypes of the *galt2galt5*, *sos5*, *fei1fei2*, *sos5fei1fei2* mutant, the quintuple mutant was also slightly hypersensitive to auxin in a root elongation assay [52] (S8 Fig).

### Altered seed coat mucilage organization in *fei2*, *sos5*, *galt2galt5*, and quintuple mutants

**Fig 8:**
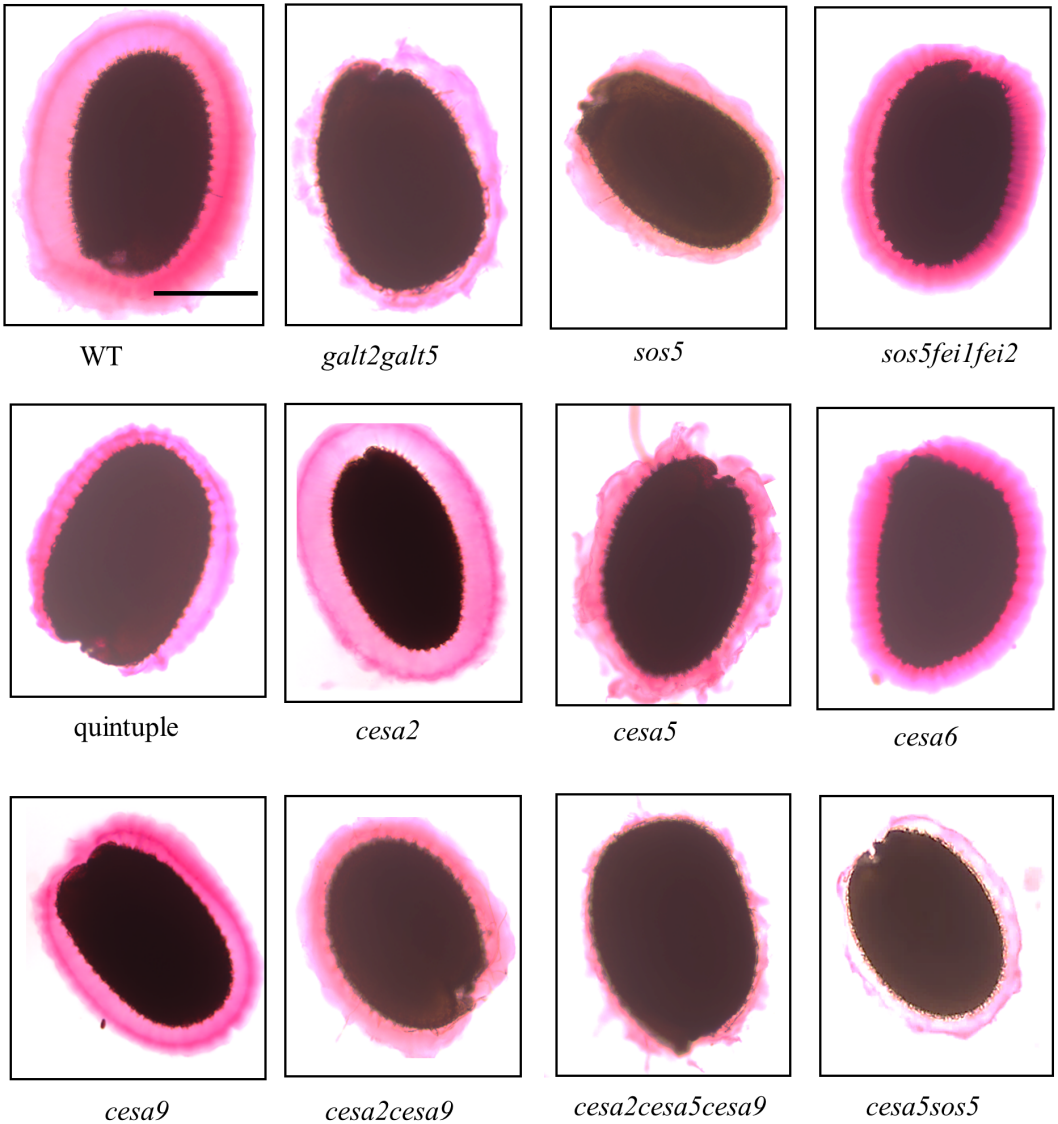
Aberrant adherent mucilage structure in the mutants as depicted by ruthenium red staining. Ten milligrams of WT and mutant seeds were hydrated in water and occasionally shaken prior to ruthenium red staining. Scale bar = 0.2 mm.

Disruption of *FEI2*, *SOS5* or *GALT2* and *GALT5* resulted in defective seed coat pectin mucilage organization [19], [20]. This prompted an examination and comparison of the mucilage in the quintuple mutant seeds with previously characterized *sos5*, *fei1fei2*, *sos5fei1fei2*, and *galt2galt5* mutants using the cationic dye ruthenium red, which stains acidic pectins. It should be noted that *fei1*, unlike *fei2*, does not show altered seed coat mucilage [20]. After removal of the non-adherent outer layer by mild shaking, a thinner inner adherent mucilage layer was observed in the mutants (Fig 8). In order to investigate the effect of cation chelators on the quintuple mutant seed coat mucilage phenotype, ruthenium red staining was conducted following treatment with the divalent cation chelator EDTA. The removal of Ca^2+^ ions reportedly leads to disruption of the ionic cross-linking of galacturonic acid residues in pectin, allowing for separation of pectin under agitation as reflected by the rapid loss of ruthenium red staining [53]. The *sos5*, *sos5fei1fei2*, and quintuple mutants, as well as the *galt2galt5* mutants albeit with less severity, displayed reduced thickness of the inner mucilage layer compared to WT (S9 Fig, S3 Table).

In order to confirm and quantify the changes in soluble versus adherent mucilage, the outer (soluble) and adherent mucilage from WT and mutant seeds were analyzed by sequential extraction of seeds with ammonium oxalate, 0.2 N NaOH, and 2 N NaOH (Table 1). The *fei2*, *sos5*, *sos5fei1fei2*, and quintuple mutants, as well as the *galt2galt5* mutants with less severity, had a significant increase in total sugars in the ammonium oxalate extract (18–35%) and the 0.2 N NaOH extract (24–55%) (i.e., soluble and weakly attached pectins) as compared with WT seeds, consistent with the increase in soluble mucilage observed by ruthenium red staining. Consistent with the above results, quintuple mutants and their parental lines displayed a decrease in total sugars in 2 N NaOH extracts, which represent the majority of adherent mucilage (i.e., strongly linked pectins and cross-linking glycans/hemicelluloses) [22], [54].

**Table 1:** Quantification of total sugars from WT, *galt2-1*, *galt5-1*, *galt2galt5*, *sos5*, *fei2*, *sos5fei1fei2*, and quintuple mucilage sequentially extracted using ammonium oxalate, 0.2 N NaOH, and 2 N NaOH.

				Genotype				
Extract	WT	*galt2-1*	*galt5-1*	*galt2 galt5*	*sos5*	*fei2*	*sos5fei1 fei2*	*galt2galt5sos5fei1fei2*
Ammonium oxalate	49±8.5^a^	50±5.5^a^	53±9.4^a^	65±11^b^	81±9.5^c^	75±11^c^	101±7.2^d^	103±8.6^d^
0.2 N NaOH	59±10^a^	61±11^a^	64±11^a^	78±8.5^b^	83±7.3^b^	79±7.1^b^	103±6.4^c^	105±6.4^c^
2 N NaOH	53±6.2^a^	46±7.7^a^	49±5.0^a^	44±3.3^a^	37±6.5^b^	39±1.3^b^	37±5.1^b^	41±5.1^a^

Intact seeds were extracted sequentially with 0.2% ammonium oxalate, 0.2 N NaOH, and 2 N NaOH, neutralized, and assayed by the phenol-sulfuric acid method against glucose standards. Analyses were performed in triplicate, and results are given as nmol/mg seed ± SE. All genotypes were grown, harvested and stored together. Different letters indicate statistically significant differences (P < 0.05) between means.

### Quintuple mutants display reduced cellulosic rays in the seed mucilage adherent layer

**Fig 9:**
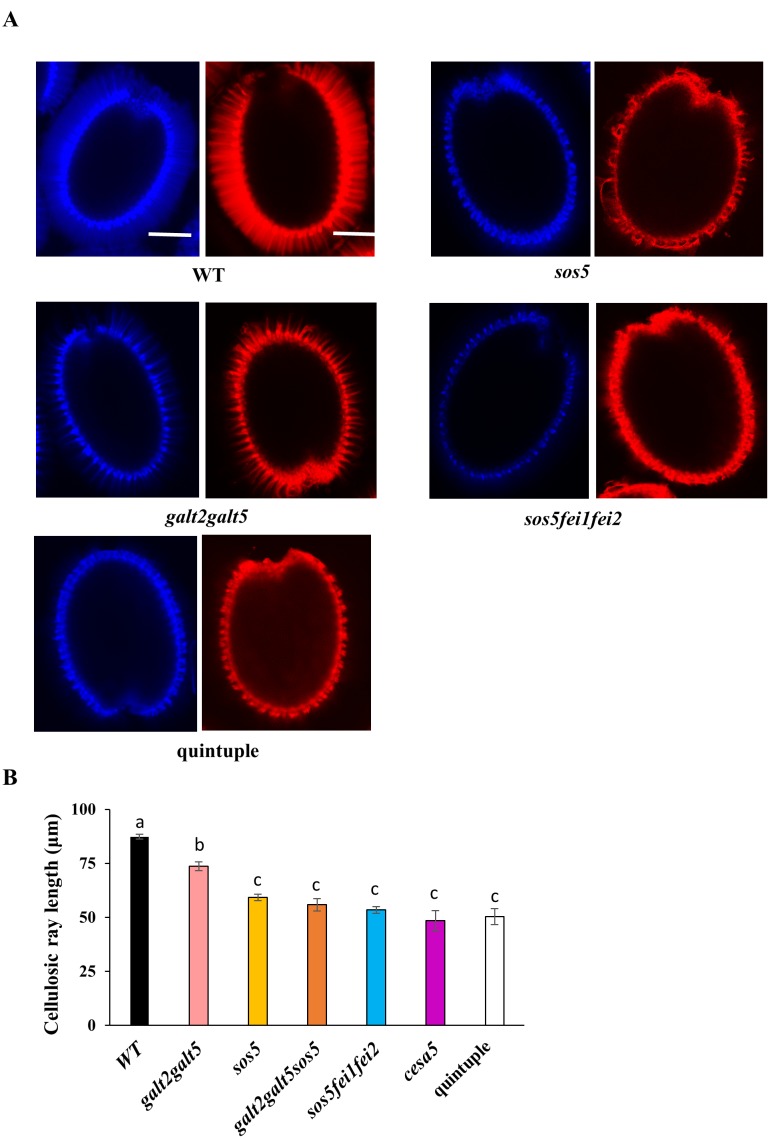
Mutants display reduced cellulosic rays in seed coat mucilage. **(A)** Calcofluor and Pontamine scarlet red staining of cellulosic rays of WT and mutant seeds was performed following gentle shaking in water. **(B)** Measurements of cellulosic ray length in WT and mutant seeds. Different letters indicate statistically significant differences (P < 0.05) between means. Error bars indicate SE of three replicates. Scale bars = 50 μm.

**Fig 10:**
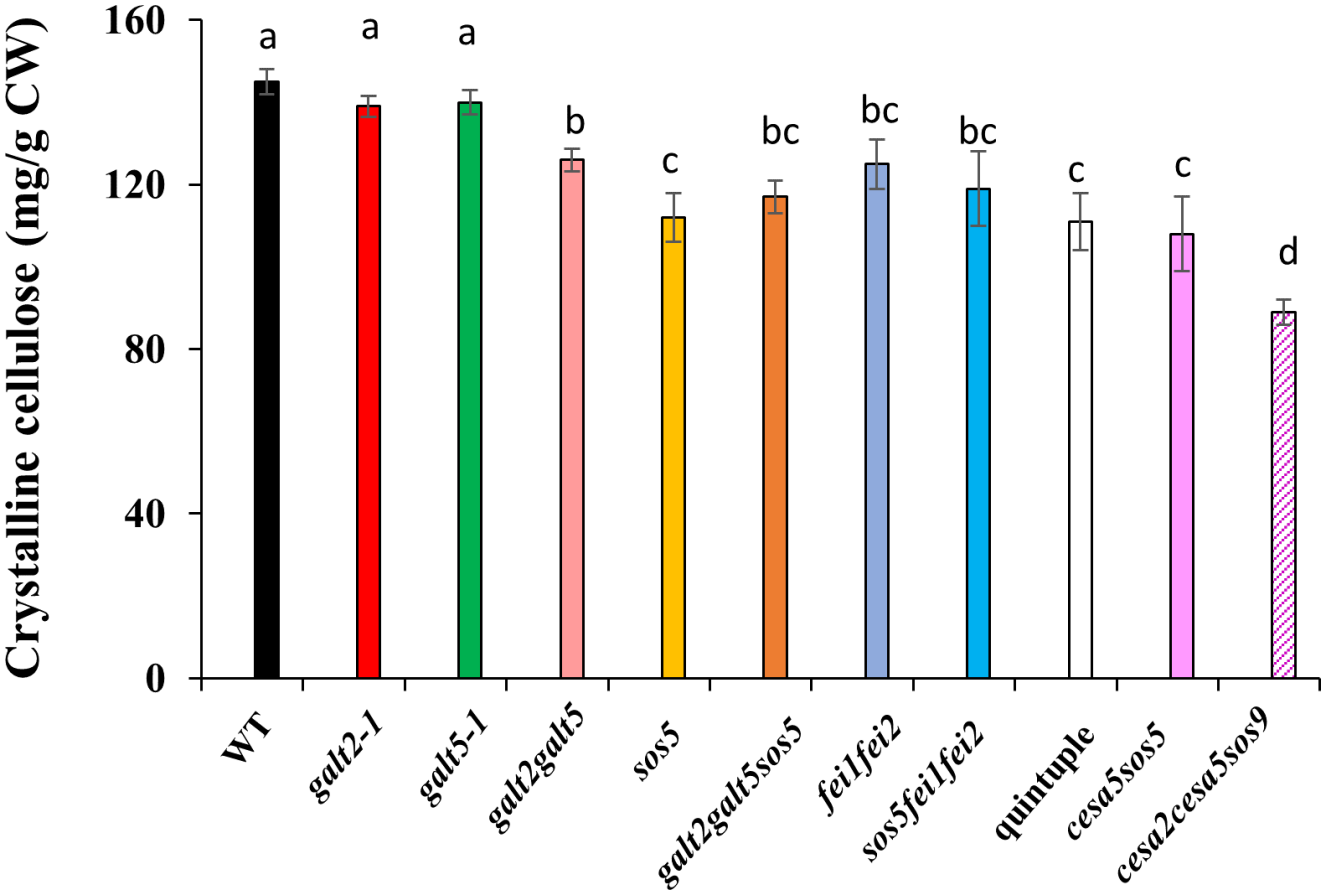
Impaired cellulosic rays in the mutants. Crystalline cellulose content determined from WT and mutant seeds. Similar results were obtained in two biological repeats. Different letters indicate statistically significant differences (P < 0.05) between means.

Seed coat cellulosic structures were visualized by staining with calcofluor, a β-glycan dye, and Pontamine Fast Scarlet S4B, a cellulose-specific dye. Cellulosic rays in quintuple mutant seeds were substantially reduced compared to WT, but similar to, *sos5fei1fei2* and *galt2galt5*, although the phenotype was somewhat less severe in *galt2galt5* (Fig 9). Crystalline cellulose content of whole seeds was also quantified (Fig 10). Consistent with the pattern observed with Pontamine staining, cellulose content in *galt2galt5* (12%), *sos5fei1fei2* (19%), and quintuple mutants (23%) were significantly reduced by 12–23% compared to WT and can be attributed to a reduction of crystalline cellulose deposition in the rays. The quintuple mutant phenotype was also compared with other known seed specific CESA mutants, namely *cesa2*, *cesa5*, and *cesa9* that are involved in secondary cell wall synthesis [55]. The decrease in crystalline cellulose from quintuple mutant seeds is less than that of *cesa2cesa5cesa9* triple mutants, indicating that the quintuple mutants likely only affect the cellulosic rays as opposed to cellulose in both the secondary cell wall and cellulosic rays, as is the case for the three CESAs [56].

Reduced seed mucilage may result in defective germination and compromised seedling establishment under conditions of reduced water potential [57], [58]. Consequently, the effect of the reduced mucilage in the quintuple mutant on seed germination was examined by growing mutant seeds in the presence of increasing concentrations of polyethylene glycol (PEG) to vary the media water potential. No significant differences were observed in the germination frequency in the quintuple mutants compared to WT at any PEG concentration, indicating that the defects in seed mucilage observed in these mutants did not affect germination in dry conditions (S10 Fig).

## Discussion

### GALT2, GALT5, SOS5, FEI1, and FEI2 act in a single genetic signaling pathway

**Fig 11:**
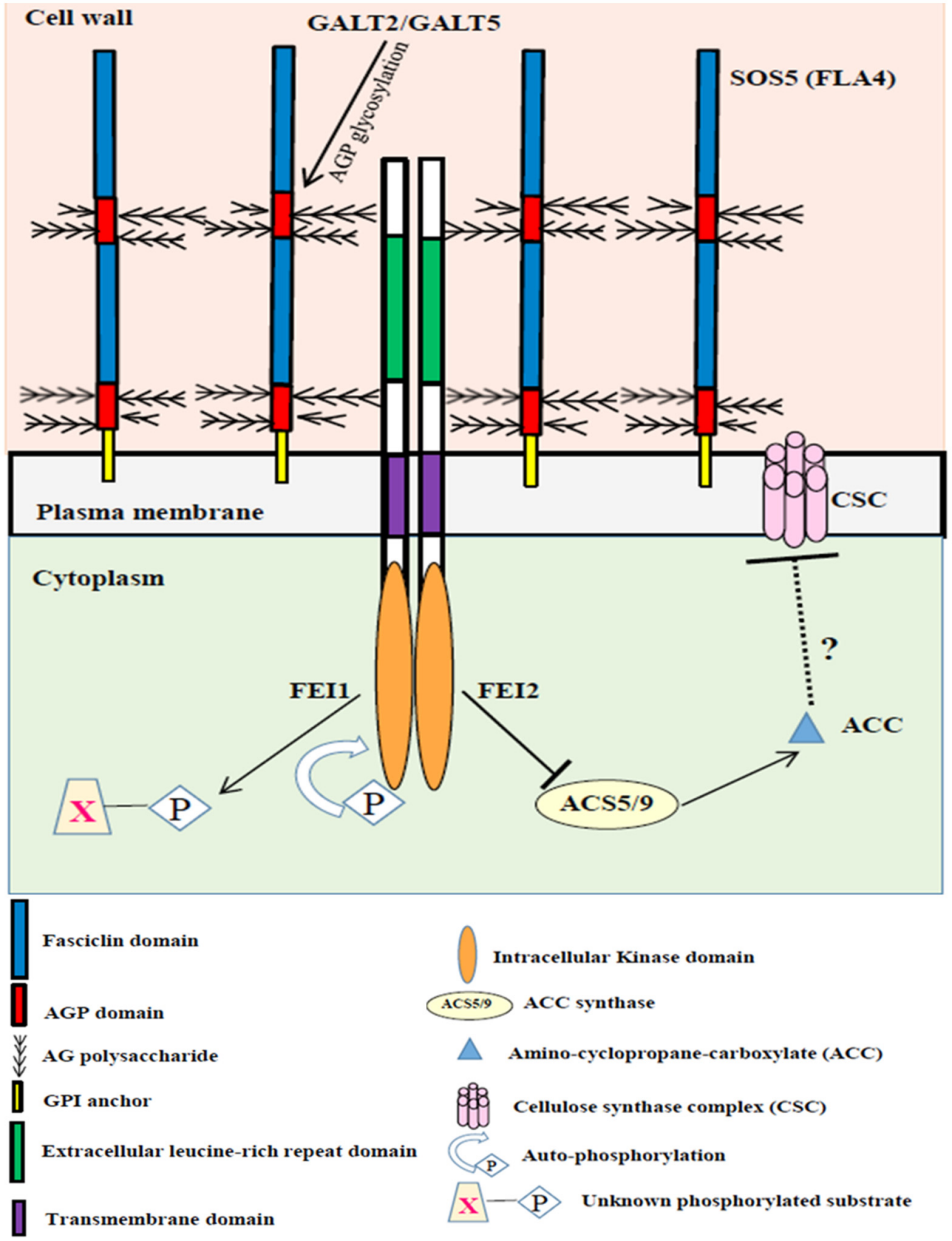
Proposed model linking GALT2 and GALT5 with SOS5 and FEI1/FEI2 in regulating cellular signaling of root growth. Signaling of normal root growth may involve GALT2/GALT5-dependent glycosylation of SOS5 and glycosylated SOS5 binding FEI1/FEI2, possibly inducing dimer formation and activation of the kinase domain as well as allowing for the binding of FEI1/FEI2 to ACC synthase. Such binding will inhibit the production of ACC, a potential signaling molecule and ethylene precursor, which directly or indirectly inhibits cellulose synthase/cellulose biosynthesis independent of ethylene. In contrast, when GALT2/GALT5-dependent SOS5 glycosylation is inhibited or SOS5 is mutated or FEI1/FEI2 is mutated, ACC synthase can no longer bind to FEI. Consequently, unbound ACC synthase produces ACC, which inhibits cellulose synthesis as well as leads to the production of ethylene.

Three separate studies in Arabidopsis have reported that loss-of-function mutants for SOS5 (a GPI-anchored fasciclin-like AGP), FEI1 and FEI2 (two cell wall receptor-like kinases), and GALT2 and GALT5 (two AGP-specific Hyp-galactosyltransferases) share similar phenotypes [18–20]. Moreover, *sos5fei1fei2* triple mutants were shown to act in a single, non-additive genetic pathway [18]. This information led to the hypothesis that these five genes act in a single linear genetic pathway, as envisioned in our proposed model (Fig 11). In order to test this hypothesis and model, quintuple mutants were generated and functionally characterized along with their parental lines, *galt2galt5* and *sos5fei1fei2*. Genetic evidence presented here indicates the five genes act in a single, non-additive genetic pathway, consistent with the model in which GALT2 and GALT5 function in the glycosylation of SOS5, which in turn interacts with FEI1/FEI2 cell wall receptor-like kinases to signal cell elongation in roots in the presence of salt or sucrose, as well as extrusion and production of seed coat mucilage. Moreover, the observation that *galt2galt5* double mutants exhibited slightly less severe root growth/swelling defects compared to the *sos5fei1fei2* triple mutants or the quintuple mutants is consistent with gene compensation by other members of the *Hyp-O-GALT* gene family [59], [19].

### SOS5-FEI1/FEI2 pathway is required for root growth and seed coat mucilage adherence

Phenotypic analysis of the knock-out quintuple mutant indicated that glycosylated SOS5 and FEI1/FEI2 are necessary for anisotropic cell expansion in Arabidopsis root cells and also play a role in cell expansion in hypocotyls of etiolated seedlings as well as in seed coat mucilage adherence. Genetic evidence coupled with biochemical analyses indicate that these genes modulate cell wall function by positively regulating the biosynthesis of cellulose, a wall component crucial for anisotropic expansion. Several receptor-like kinases (RLKs) were identified that likely act as sensors of cell wall integrity, regulating cell growth in response to cell wall perturbations [52], [60]. In Arabidopsis, there are approximately 600 receptor-like kinases (RLKs), several of which act in a variety of signaling pathways that function in plant development [61]. Two other well-documented receptor kinases implicated in regulating cell wall function include the wall-associated kinases (WAKs) and four members of the *Catharanthus roseus* RLK1-Like kinases (CrRLK1L) [62–64]. The four members include FERONIA (FER), THESEUS1 (THE1), HERCULES1 (HERK1), and HERK2. WAKs are implicated to function in cell expansion and in cell wall signaling in response to pathogen and stress [65], [66]. Interestingly, *wak2* knock-out mutants also display impaired cell expansion in root, but unlike *fei1fei2* mutants, they exhibit a dependence on sugars and salts for seedling growth [67]. THESEUS1 (THE1) RLK acts as a suppressor of *cesA6*, which suggests that it functions in sensing and regulating cell wall integrity [60]. In contrast, disruption of the two partially redundant *FEI1* and *FEI2* RLKs was found to cause root growth arrest, root tip swelling, and a reduction in crystalline cellulose production in root tips when grown under restrictive conditions of either elevated sucrose or salt [18].

One potential ligand for the FEI1 and FEI2 RLKs is the extracellular FLA4, SOS5, which is likely glycosylated by the action of GALT2 and GALT5. Several lines of evidence indicate that glycosylated SOS5 functions by interacting with FEI1/FEI2 either directly or indirectly: 1) the patterns of expression of *GALT2*, *GALT5*, *SOS5*, *FEI1*, and *FEI2* and are largely overlapping, 2) all these mutants have a very similar root-elongation phenotype in response to salt and sucrose, 3) all the mutants exhibit nearly identical radial swelling of root tips as well as wider hypocotyls, 4) all the mutants show a substantial reduction of cellulose biosynthesis in the root tip region as well as reduced cellulosic rays in the seed coat mucilage layer, 5) all the mutants demonstrate suppression of radial swelling by inhibitors of ethylene biosynthesis by AVG, AIB and CoCl_2_, but not by genetic disruption of ethylene perception by silver nitrate, 6) all the mutants display ectopic lignin deposition in the roots, 7) the effect of salt and sucrose on these mutants was not an outcome of increased osmotic potential of the medium, 8) all the mutants have aberrant non adherent mucilage extrusion, and 9) double, triple and quintuple mutants displayed non-additive phenotypes, providing genetic evidence which indicates these genes and their encoded proteins, regulate cell expansion in a linear pathway.

### Role of ethylene and ABA in the GALT2/GALT5/SOS5/FEI1/FEI2 pathway

Mounting lines of evidence support the link between ethylene and several mutants that affect root growth anisotropy, including *sabre*, *cev1*, and *lue1* [48], [49], [68]. But unlike *fei1fei2*, the swollen root tip phenotype of these mutants can be rescued by disrupting ethylene signaling and/or ethylene action [48], [49]. In the root, ethylene strongly inhibits root elongation, but radial expansion is only modestly increased and microtubules appear to be unaffected [69]. Thus, in the root, ethylene appears to act primarily by inhibiting the overall amount of cell expansion, not its orientation. In addition, Xu et al. demonstrated that the cytoplasmic domain of FEI2 interacts with ACC synthase (ACS2/5), leading to the hypothesis that SOS5 and the FEIs might act in a linear genetic pathway that depends on ACC, but not on ethylene signaling, upstream of cellulose deposition [18]. This indicates that swelling in the absence of *FEI1*/*FEI2*, *GALT2/GALT5*, or *SOS5* depends on a hitherto undiscovered pathway for ethylene perception or that ACC itself acts as a signaling molecule. Reduced root growth of *gat2galt5*, *sos5fei1fei2*, and the quintuple mutant in response to IAA indicates that auxin is required for the radial cell expansion that occurs in response to decreases in cellulose synthesis in the absence of functional GALT2, GALT5, SOS5, FEI1, and FEI2 proteins (S8 Fig).

### Mechanisms of action involved with the GALT2/GALT5/SOS5/FEI1/FEI2 pathway

Glycosylated SOS5 is required for stimulating the SOS5/FEI signaling pathway in roots tips and in the adherent mucilage layer of seed coat. The interaction may be direct or indirect and mediated by other proteins or cell wall components. One potential mechanism explaining the phenotype observed when the GALT2GALT5/SOS5/FEI1FEI2 pathway is disrupted in roots can be attributed to the elevated levels of ROS in the elongation zone of Arabidopsis roots in response to ACC. Such elevated ROS levels lead to the cross-linking of Hyp-rich glycoproteins and callose deposition in the cell wall, both of which may contribute to reduced cell expansion [70]. Related to this, Xue and Seifert [71] demonstrated that *sos5* mutants indeed exhibit production of higher levels of ROS compared to WT plants.

An abundant set of proteins for the biosynthesis and modification of polysaccharides involved in seed coat mucilage synthesis, secretion, modification, and stabilization have been identified [35–38], [53–58]. Both CESA5 and SOS5 have been proposed to facilitate cellulose-mediated seed coat mucilage adherence [20], [55], [56]. Initial phenotypic analysis by Harpaz-Saad et al. [20] suggested that SOS5 may regulate mucilage adherence by influencing CESA5 function. However, detailed characterization of *sos5* and *cesa5* single and double mutants by Griffiths et al. [35] and Francoz et al. [72] indicated that SOS5 mediates mucilage adherence independently of CESA5, mostly by influencing pectins. A role for pectin in establishing cellulose microfibril orientation was previously suggested by Yoneda et al. [73] during cell expansion. A similar mechanism may exist in case of mucilage biosynthesis, which occurs after cell expansion. In any event, based on the work presented here, it is proposed that glycosylation of SOS5 is critical for its function. A more complete understanding of mucilage secretory cell differentiation and establishment will require additional genetic and biochemical characterization of the many key genes and proteins already associated with this model system, along with new ones yet to be discovered [37].

Although SOS5 is proposed to act as a ligand for FEI1/FEI2; further experiments are needed confirm biochemical interaction between the extracellular domains of FEIs with (glycosylated) SOS5. Although there is no direct evidence of glycosylation of SOS5 by GALT2 and GALT5, β-Yariv precipitable AGPs examined from *galt2galt5* mutants revealed that glycosylation of virtually all AGPs, as opposed to a single or subset of these AGPs, was affected [19]. The proposed model for the GALT2GALT5/SOS5/FEI1FEI2 pathway provides a basis for testing and manipulating the signaling process to better understand and enhance root growth (Fig 11). In such a model, AGPs, may bind an unknown signaling ligand or may directly act as plasma membrane/cell wall pressure sensor to relay information to FEI1/FEI2. Indeed, AGPs are suggested to form an electrostatic cushion between the relatively rigid cell wall and the plasma membrane [74], [75]. Thus, GPI-anchored AGPs such as SOS5 could effectively act as a CWI sensor by perceiving and signaling differences in turgor pressure when subjected to elevated salt or sucrose. Alternatively, the reduced glycosylation of SOS5 may result in less SOS5 being deposited in the plasma member to function in this pathway, since glycosylation of AGPs is reported to facilitate their secretion [76].

### Conclusions

This study corroborates, links, and extends previous biochemical and genetic studies designed to understand the roles of GALT2/GALT5, SOS5, and FEI1/FEI2 [17–20]. The current work provides genetic evidence for all these proteins interacting in a single, linear pathway. Moreover, this work provides evidence for the important role of AG polysaccharides in the function of SOS5. This function likely involves the ability of glycosylated SOS5 to sense turgor pressure and/or CWI and transmit that information to the FEI1/FEI2 RLK system in order to regulate cell wall synthesis by stimulating cellulose synthase. Indeed, both Arabidopsis root growth and seed coat mucilage adherence provide useful developmental models where such a signaling system appears to operate.

## Supporting Information

S1 Fig Transcript profiling of *GALT2*, *GALT5*, *FEI1*, and *FEI2* throughout different developmental stages in Arabidopsis as depicted by PlaNet. Y-axis: expression values, x-axis: tissues/treatments, red dots: average expression, green dots: expression from individual microarray/RNAseq experiments. The horizontal red lines indicate the expression patterns during root and seed development.
(TIF)

S2 Fig Expression patterns of well characterized representative genes through the course of seed development. These expression patterns were mined from publicly accessible database Bio-Array Resource eFP browser [Winter D et al (2007)], which is based on the microarray analysis of laser-captured micro-dissected seeds [33].
(TIF)

S3 Fig Generation of quintuple knock-out mutants. **(A)** Gene structures and T-DNA insertion sites for *SOS5*, *FEI1*, and *FEI2*. (**B**) RT-PCR analysis of *galt2galt5sos5fei1fei2* quintuple mutants to confirm null status. Total RNA was extracted from rosette leaves of 2-week-old WT and homozygous mutant plants of the indicated genotypes. *UBQ10* was used as the loading control. Experiments were repeated at least twice with virtually identical results. Arrows in **(A)** indicate the locations of primers used for RT-PCR.
(TIF)

S4 Fig Hypocotyl phenotype of the quintuple mutants. **(A)** Quantification of hypocotyl widths from WT and mutant seedlings grown for four days in the dark on MS medium with 0% sucrose. Values represent the mean (n = 10) ± SE. Different letters indicate statistically significant differences (P < 0.05) between means. (**B**) Representative images of hypocotyls from WT and mutants of the indicated genotypes. Scale bar = 1 mm.
(TIF)

S5 Fig Cell elongation is affected in the mutants under restrictive conditions. **(A)** Root meristem cell numbers in WT and mutant seedlings were determined 5 d after transfer to the indicated treatment. None of the values are significantly different from one another. **(B)** Root epidermal cell lengths from the zone of elongation in WT and mutants were measured 5 d after transfer to the indicated treatment. The cell number and cell length data are averages of two biological replicates, with each replicate having 10 seedlings and n = 30 cells/per seedling and error bars representing ±SD. Different letters indicate statistically significant differences (P < 0.05) between means.
(TIF)

S6 Fig Root elongation in response to mannitol. Four-day-old WT and mutant seedlings were grown on vertical MS agar plates and were transferred to MS agar plates supplemented with 300 mM mannitol. The plates were placed vertically, and the root tip of each seedling was marked. Root elongation was measured 7, 14, and 21 d after transfer. Error bars are SE of three replicates; n = 20.
(TIF)

S7 Fig Salt hypersensitivity assessed by the root bending assay. **(A)** Five-day-old seedlings grown on MS plates were transferred to MS plates with 100 mM NaCl and reoriented at an angle of 180° (upside down). Images were taken 5 d after seedling transfer. (**B**) Analysis of root curvature of the indicated seedlings was measured using ImageJ software. Values represent the mean (n = 5) ± SE. Different letters indicate statistically significant differences (P < 0.05) between means. Scale bar = 10 mm.
(TIF)

S8 Fig Quantification of root elongation of WT and mutants in response to varying concentrations of auxin. Five-day-old seedlings were transferred to media containing the indicated auxin concentration, and the roots were grown for five days in the supplemented media before performing measurements. Values represent the mean ± SE (n>10). Different letters indicate statistically significant differences (P < 0.05) between means.
(TIF)

S9 Fig Effect of the cationic chelator EDTA on seed mucilage phenotype. WT and mutant seeds were stained with ruthenium red for pectin following pre-treatment with 50 mM EDTA and gentle shaking. Scale bar = 0.25 mm.
(TIF)

S10 Fig Germination frequencies for WT and mutant seeds on media containing increasing concentrations of polyethylene glycol (PEG). Germination percentages were determined from three independent experiments with more than 200 seeds per line for each experiment. Values are means ± SE. None of the values were significantly different from one another.
(TIF)

S1 Table Primers used in this study. (DOCX)

S2 Table List of candidate genes coexpressed with *FEI1* using the Gene CAT coexpression tool. (DOCX)

S3 Table Comparison of the key parameters of adherent mucilage from mutant seed coats. (DOCX)
