# Supplementary figures and images for: Glycosylation of a Fasciclin-Like Arabinogalactan-Protein (SOS5) Mediates Root Growth and Seed Mucilage Adherence via a Cell Wall Receptor-Like Kinase (FEI1/FEI2) Pathway in Arabidopsis
Source: PLoS One. 2016 Jan 5;11(1):e0145092. doi: 10.1371/journal.pone.0145092 (PMC4701510; doi:10.1371/journal.pone.0145092)

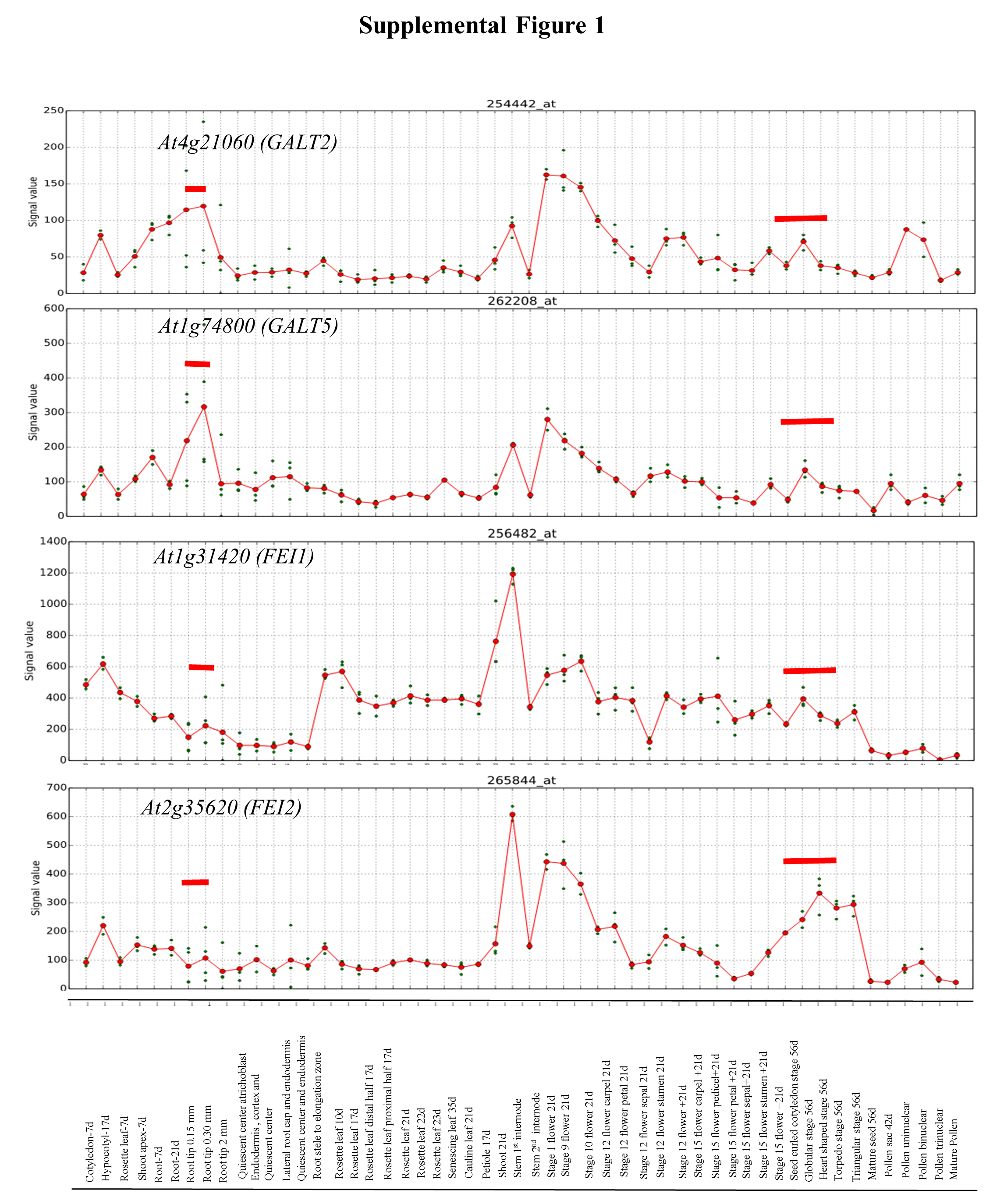

Supplement: S1 Fig — Y-axis: expression values, x-axis: tissues/treatments, red dots: average expression, green dots: expression from individual microarray/RNAseq experiments. The horizontal red lines indicate the expression patterns during root and seed development. (TIF) [file pone.0145092.s001.tif]

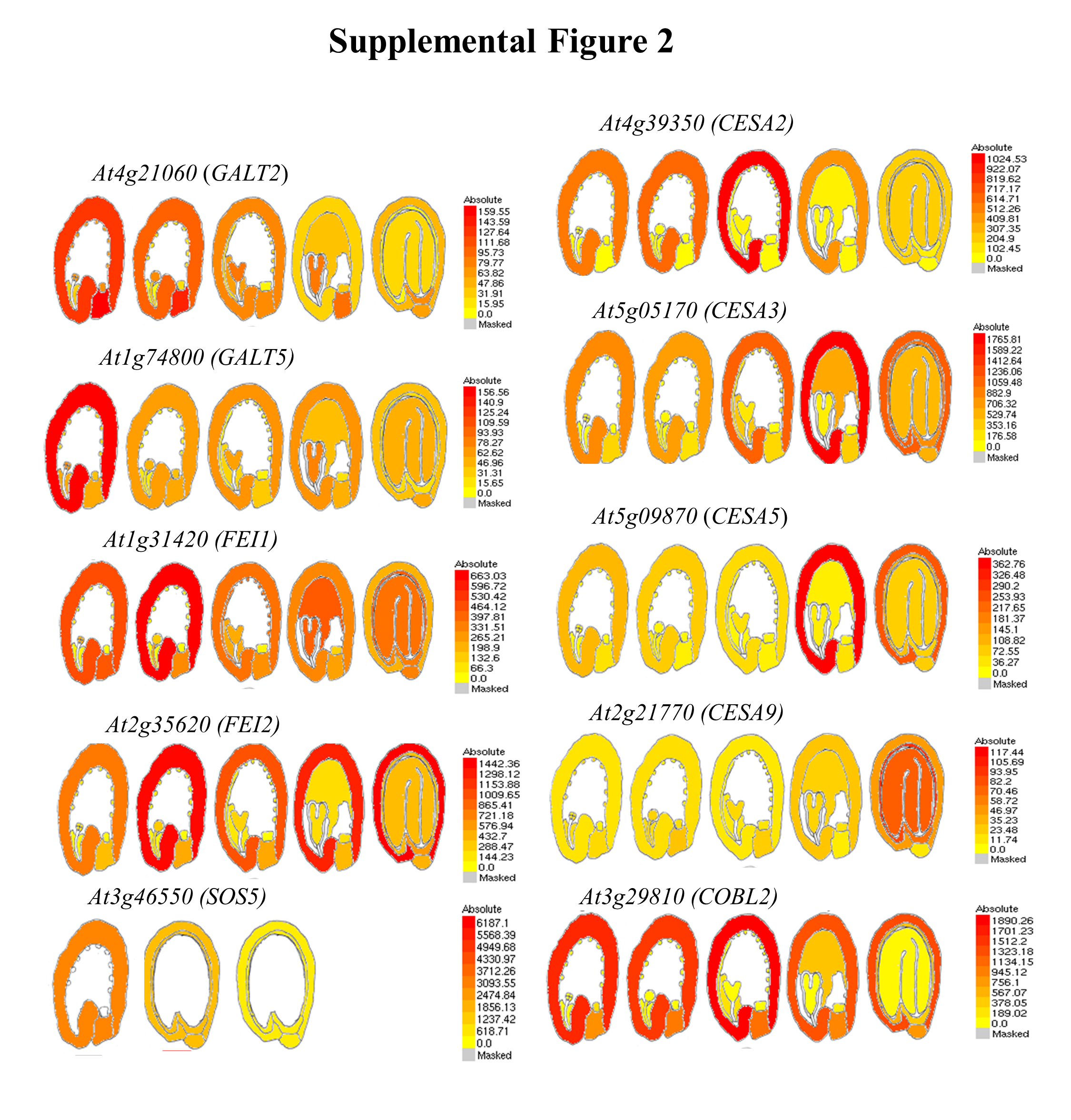

Supplement: S2 Fig — These expression patterns were mined from publicly accessible database Bio-Array Resource eFP browser [Winter D et al (2007)], which is based on the microarray analysis of laser-captured micro-dissected seeds [33]. (TIF) [file pone.0145092.s002.tif]

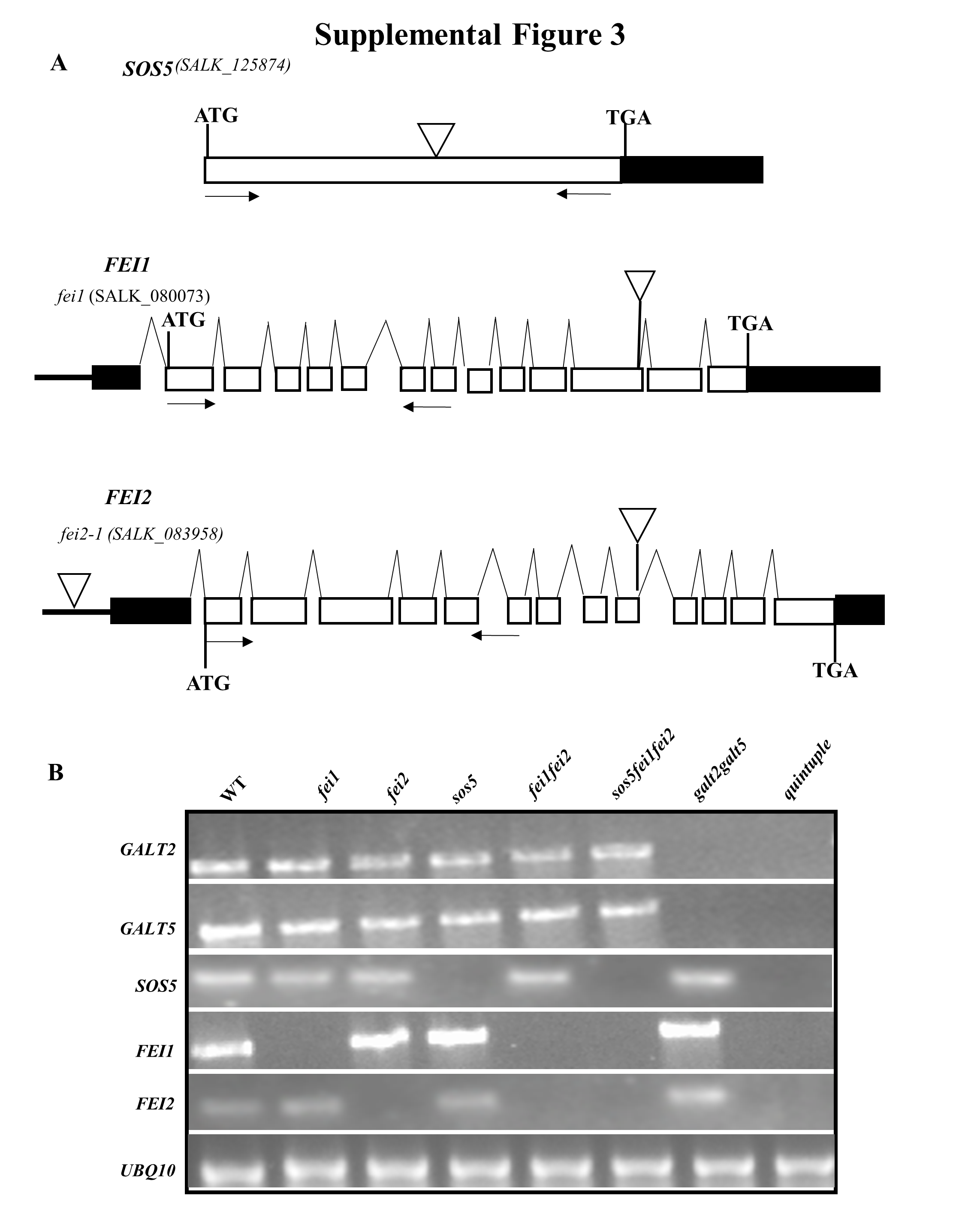

Supplement: S3 Fig — (A) Gene structures and T-DNA insertion sites for SOS5, FEI1, and FEI2. (B) RT-PCR analysis of galt2galt5sos5fei1fei2 quintuple mutants to confirm null status. Total RNA was extracted from rosette leaves of 2-week-old WT and homozygous mutant plants of the indicated genotypes. UBQ10 was used as the loading control. Experiments were repeated at least twice with virtually identical results. Arrows in (A) indicate the locations of primers used for RT-PCR. (TIF) [file pone.0145092.s003.tif]

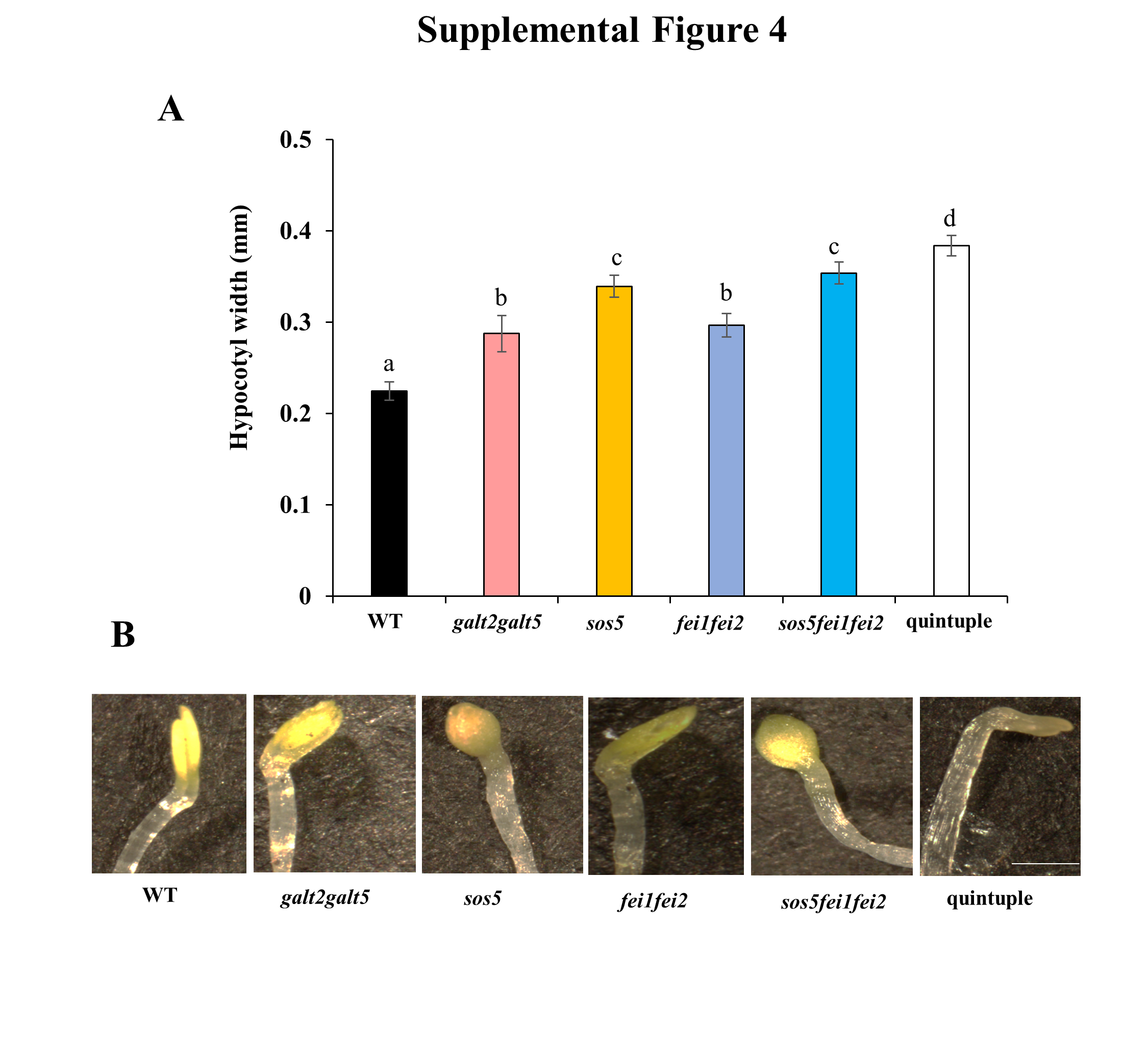

Supplement: S4 Fig — (A) Quantification of hypocotyl widths from WT and mutant seedlings grown for four days in the dark on MS medium with 0% sucrose. Values represent the mean (n = 10) ± SE. Different letters indicate statistically significant differences (P < 0.05) between means. (B) Representative images of hypocotyls from WT and mutants of the indicated genotypes. Scale bar = 1 mm. (TIF) [file pone.0145092.s004.tif]

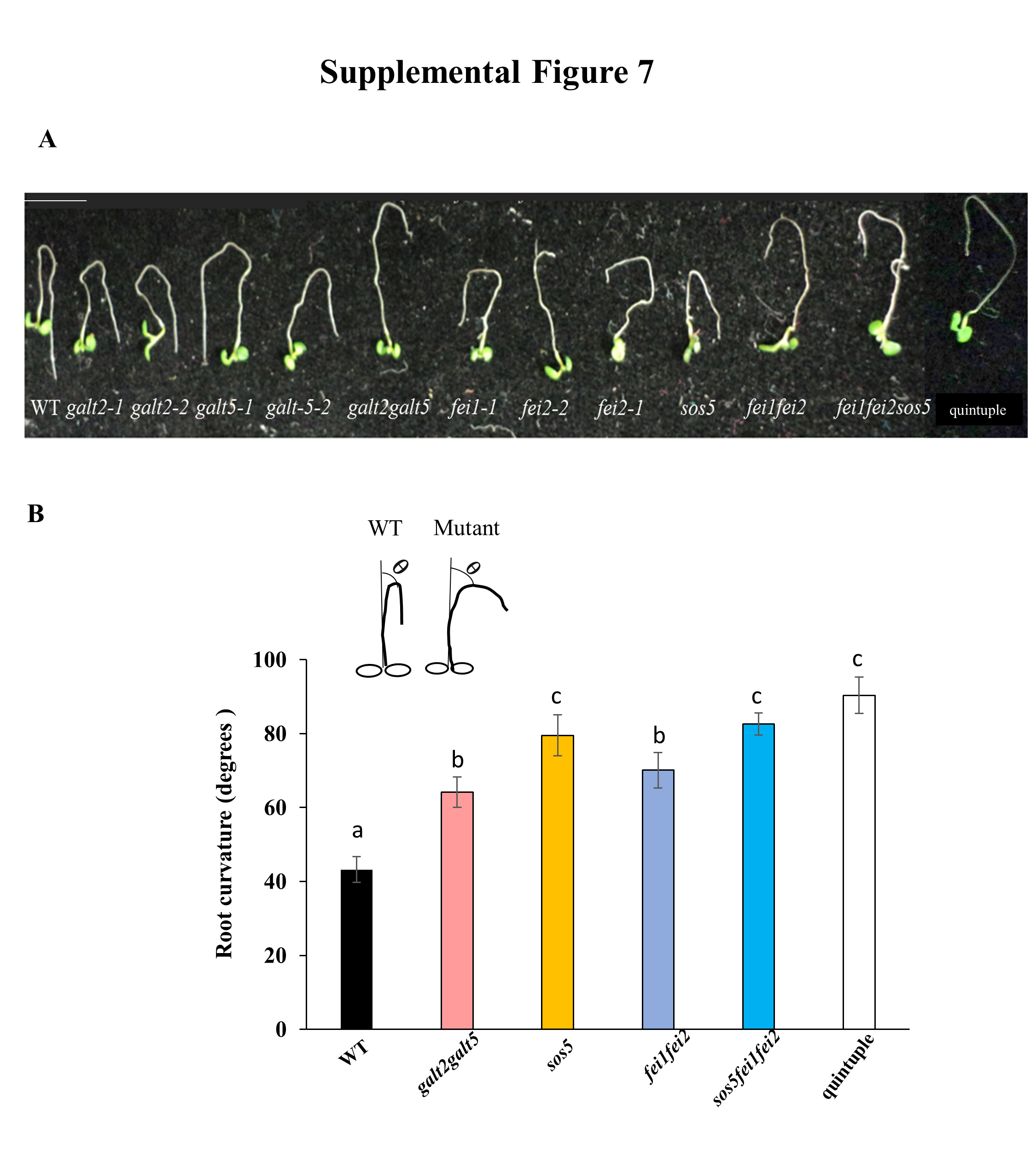

Supplement: S7 Fig — (A) Five-day-old seedlings grown on MS plates were transferred to MS plates with 100 mM NaCl and reoriented at an angle of 180° (upside down). Images were taken 5 d after seedling transfer. (B) Analysis of root curvature of the indicated seedlings was measured using ImageJ software. Values represent the mean (n = 5) ± SE. Different letters indicate statistically significant differences (P < 0.05) between means. Scale bar = 10 mm. (TIF) [file pone.0145092.s007.tif]

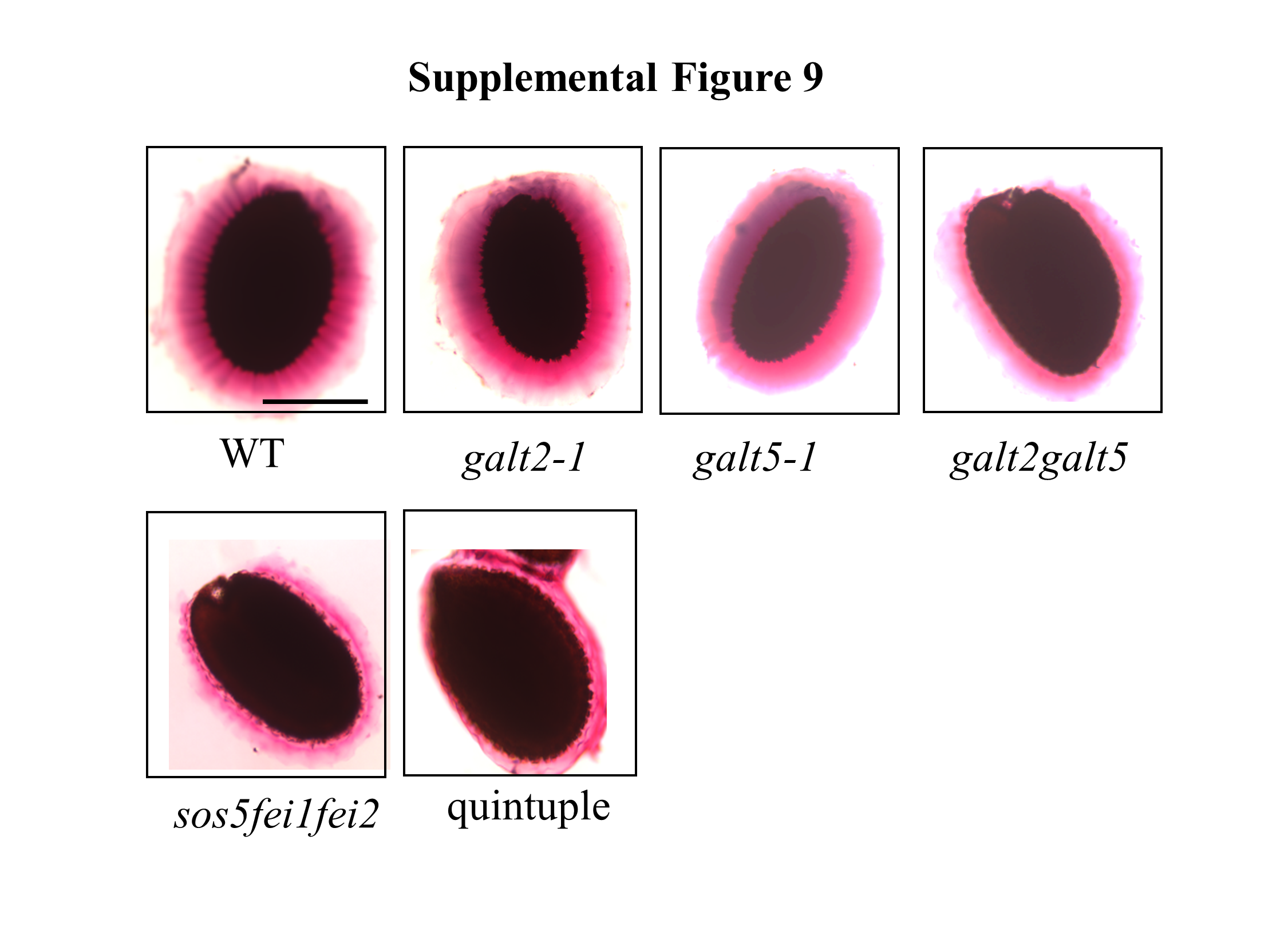

Supplement: S9 Fig — WT and mutant seeds were stained with ruthenium red for pectin following pre-treatment with 50 mM EDTA and gentle shaking. Scale bar = 0.25 mm. (TIF) [file pone.0145092.s009.tif]
