# Supplementary material for: Glycosylation of a Fasciclin-Like Arabinogalactan-Protein (SOS5) Mediates Root Growth and Seed Mucilage Adherence via a Cell Wall Receptor-Like Kinase (FEI1/FEI2) Pathway in Arabidopsis
Source: PLoS One. 2016 Jan 5;11(1):e0145092. doi: 10.1371/journal.pone.0145092 (PMC4701510; doi:10.1371/journal.pone.0145092)
Supplement: S1 Table — (DOCX) [file pone.0145092.s011.docx]

**S1 Table. Primers used in this study.**

| **Purpose** | **Forward** | **Reverse** | |  |
| --- | --- | --- | --- | --- |
| **Screening of homozygous mutants** | | | | |
| *GALT2* | galt2-1RP-  TCACTTGGTCATTCCCTTTTG | galt2-1LP-  CAAATCGATGGAGTCTCTCCA | |  |
| *GALT5*  Lba1.3 | galt5-1RP-TTTCCACTTTCGACAATTTGG  ATTTTGCCGATTTCGGAAC | GALT5-1LP-  CTAATTACATGGTTTTGCGGG | |  |
| *SOS5* | SOS5-2RP-  CACCATGGCCGCCGCAATTAACGTCACC | SOS5-2LP-  GCCGGAAGAAACTATCTCACGC | |  |
| *FEI1*  T-DNA left border | FEI1-1-RP  GAAGCTGGAAATGTTGAATGA  AGAGGCAATCAGCTGTTGCCCGTCTCACTGGTG | FEI1-LP-TTAATCAGAGCTGGAATCATAAAATTC | |  |
| *FEI2*  T-DNA left border | FEI2-1-RP  ACAAATCGATATTGTGTGCAATGACAG  TTACCCAACTTAATCGCCTTGCAGCACA | FEI2-1-LP  TCAATCGGAGCTGGAGTCGTAGAAG | |  |
| **RT-PCR** |  |  | |  |
| *GALT2* | RTF-  TCTTTGTTGCACTTAATCCAAGAAG | RTR-  TGTGGTCGACCTTTCAACAAATTAT | |  |
| *GALT5*  *FEI1*  *FEI2* | RTF-  TATGTGAACACGGAGCTCTTGCATTC  RTF-  ATATGGAGCAATACCTACAGC  RTF- GAAACTGGAATCTCTTAATGAAGAGC | RTR-TCCATCTTGAACAGCCGTAATTTATGTCT  RTR-  TGATGCGCTAATCAGCAGCTTACCAG  RTR-  GGTCGACCTTTCAACAAATTAT | |  |
| *SOS5*  *UBQ10* | RTF-  CACCATGGCGAACGTAATCTC  AATTTCC  GTCGACCCTTCACTTGGTGT | RTR-TACCAAAACATAACAAAATGCTATAC  ATCCTCAAGCTGCTTTCCAG | |  |
| **Q-PCR**  *GALT2*  *GALT5*  *SOS5* | CATAAGCTTAGGCTATTCAA  GATGGACATAAATTACGGCTGTTCAAGATGGA  TCGGAGTATCCAAAGTTCTTTTG | GGTCGACCTTTCAACAAATTAT  TCTTAAGAGCTTATCCCATAAGCATA  TCATACCAAAACATAACAAAATGCTA | |  |
| *FEI1* | AATCACGAAAAACGGCCAAGGGATA | | TCAATCGGAGCTGGAGTCGTAGAAGT | |
| *FEI2* | TAAACTTCTTAATCAGCGAAAACCGGG | | TTAATCAGAGCTGGAATCATAAAATTCG | |
| *GAPC* | TCAGACTCGAGAAAGCTGCTAC | | GATCAAGTCGACCACACGG | |
