# Supplementary material for: Glycosylation of a Fasciclin-Like Arabinogalactan-Protein (SOS5) Mediates Root Growth and Seed Mucilage Adherence via a Cell Wall Receptor-Like Kinase (FEI1/FEI2) Pathway in Arabidopsis
Source: PLoS One. 2016 Jan 5;11(1):e0145092. doi: 10.1371/journal.pone.0145092 (PMC4701510; doi:10.1371/journal.pone.0145092)
Supplement: S2 Table — (DOCX) [file pone.0145092.s012.docx]

**S2 Table. List of candidate genes coexpressed with *FEI1* using the Gene CAT coexpression tool.**

| **Gene Name** | **Predicted function** | **Pearson’s correlation coefficient** |
| --- | --- | --- |
| *At1g31420 (FEI1)* | | |
| *At2g35620* | cell wall receptor like kinase (FEI2) | 0.73940 |
| *At5g09870* | cellulose synthase (CESA5) | 0.70353 |
| *At4g32410* | cellulose synthase (CESA1) | 0.60589 |
| *At5g05170* | cellulose synthase (CESA3) | 0.56805 |
| *At5g64740* | cellulose synthase (CESA6) | 0.52679 |
| *At4g39350* | cellulose synthase (CESA2) | 0.44579 |
| *At4g26940* | galactosyltransferase protein GT31 family | 0.34069 |
| *At3g06440* | galactosyltransferase protein GT31 family (GALT3) | 0.35562 |
| *At1g53290* | galactosyltransferase protein GT31 family | 0.19700 |
| *At1g74800* | galactosyltransferase protein GT31 family (GALT5) | 0.16268 |
| *At1g05170* | galactosyltransferase protein GT31 family | 0.14670 |
| *At2g32430* | galactosyltransferase protein GT31 family | 0.14442 |
| *At5g53340* | galactosyltransferase protein GT31 family (HPGT1) | 0.07022 |
| *At4g32120* | galactosyltransferase protein GT31 family (HPGT2) | 0.05157 |
| *At1g11730* | galactosyltransferase protein GT31 family | 0.00675 |
| *At5g55730* | fasciclin-like arabinogalactan protein (FLA1) | 0.34251 |
| *At4g12730* | fasciclin-like arabinogalactan-protein (FLA2) | 0.54985 |
| *At2g45470* | fasciclin-like arabinogalactan-protein (FLA8) | 0.54608 |
| *At5g44130* | fasciclin-like arabinogalactan-protein (FLA13) | 0.48029 |
| *At1g03870* | fasciclin-like arabinogalactan-protein (FLA9) | 0.45305 |
| *At2g04780* | fasciclin-like arabinogalactan-protein (FLA7) | 0.36382 |
| *At5g60490* | fasciclin-like arabinogalactan-protein (FLA12) | 0.19956 |
| *At2g23130* | arabinogalactan-protein (AGP17) | 0.37150 |
| *At2g46330* | arabinogalactan-protein (AGP16) | 0.19776 |
| *At1g02730* | ATCSLD5, cellulose synthase like | 0.13064 |
| *At5g65390* | arabinogalactan-protein (AGP7) | 0.14295 |
| *At5g10430* | arabinogalactan-protein (AGP4) | 0.11422 |
| *At2g15390* | fucosyltransferase (FUT4) | 0.01729 |
