# Supplementary material for: Glycosylation of a Fasciclin-Like Arabinogalactan-Protein (SOS5) Mediates Root Growth and Seed Mucilage Adherence via a Cell Wall Receptor-Like Kinase (FEI1/FEI2) Pathway in Arabidopsis
Source: PLoS One. 2016 Jan 5;11(1):e0145092. doi: 10.1371/journal.pone.0145092 (PMC4701510; doi:10.1371/journal.pone.0145092)
Supplement: S3 Table — (DOCX) [file pone.0145092.s013.docx]

**S3 Table. Comparison of the key parameters of adherent mucilage from mutant seed coats.**

| **Genotype** | **Mass (μg)** | **Size (mm^2^)** |
| --- | --- | --- |
| WT | 1.82 + 0.06 | 0.51 + 0.09^a^ |
| *galt2galt5* | 1.71 + 0.02 | 0.40 + 0.05^b^ |
| *sos5* | 1.75 + 0.03 | 0.30 + 0.02^c^ |
| *fei2* | 1.73 + 0.04 | 0.35 + 0.02^c^ |
| *sos5fei1fei2* | 1.79 + 0.03 | 0.32 + 0.04^c^ |
| *galt2galt5sos5* | 1.75 + 0.03 | 0.29 + 0.05^d^ |
| *galt2galt5sos5fei2* | 1.74 + 0.04 | 0.27 + 0.03^d^ |

The mass and size values denote an average of 100 seeds, and these analyses were done it duplicate. Different letters indicate statistically significant differences (P < 0.05) between means. The measurement of adherent mucilage was performed using the method described by Yu et al. [1].

[1] Yu L, Shi D, Li J, Kong Y, Yu Y, Chai G, Hu R, Wang J, Hahn M G and Zhou G. (2004) CELLULOSE SYNTHASE-LIKE A2, a glucomannan synthase, is involved in maintaining adherent mucilage structure in Arabidopsis seed. Plant Physiol. 164: 1842–1856.
